# Criminal minds in dementia: A systematic review and quantitative meta-analysis

**DOI:** 10.1038/s41398-025-03523-z

**Published:** 2025-08-28

**Authors:** Matthias L. Schroeter, Marija Žuvela, Lena Szabo

**Affiliations:** 1 Max Planck Institute for Human Cognitive and Brain Sciences, Stephanstr. 1A, 04103 Leipzig, Germany; 2 University Hospital Leipzig, Clinic for Cognitive Neurology, Liebigstr. 16, 04103 Leipzig, Germany; 3 Institute for Interdisciplinary Studies, University of Amsterdam, Science Park 904, 1098 XH Amsterdam, The Netherlands; 4 University of Sydney, Brain and Mind Centre, 94 Mallett St, Camperdown NSW 2050, Sydney, Australia; 5 Institute of Systems Neuroscience, University Medical Center Hamburg-Eppendorf, Martinistr. 52, 20251 Hamburg, Germany

**Keywords:** Psychiatric disorders, Scientific community

## Abstract

**Introduction:**

Subjects with dementia might exhibit criminal risk behavior (CB), even in early disease stages.

**Methods:**

This systematic review and quantitative meta-analysis investigated CB prevalence across all neurodegenerative syndromes according to PRISMA criteria and preregistered in PROSPERO. Mean frequencies and odds ratios were calculated and compared.

**Results:**

Finally, the meta-analysis included 14 studies with 236,360 persons. Studies originated from different countries, with largest contributing country being the U.S.A., followed by Scandinavian countries, i.e., Sweden and Finland, and additionally Germany and Japan. All quantitative analyses revealed a very consistent picture: CB prevalence was highest in behavioral variant frontotemporal dementia ( >50%), followed by semantic variant primary progressive aphasia (40%), but rather low in vascular dementia and Huntington’s disease (15%), Alzheimer’s disease (10%), lowest in Parkinsonian syndromes ( <10%). The systematic literature review revealed that CB prevalence is more frequent in early disease course than in the general population, but declines thereafter below population levels. Men are overrepresented.

**Discussion:**

CB is a common symptom in dementia, most pronounced in frontotemporal dementia. CB committed for the first time at mid-age could be an indicator of incident dementia, requiring earliest diagnosis and therapy. As present studies show a wide variability in assessment methods and cohorts investigated, and had been conducted in a minority of countries world-wide, large prospective international studies are warranted that systematically apply homogeneous methods and standardized questionnaires in assessing criminal risk behavior in different dementia syndromes.

## Introduction

Neurodegenerative diseases might affect several functions, ranging from memory in Alzheimer’s disease (AD) to behavior, such as in behavioral variant frontotemporal dementia (bvFTD), language function in the so called primary progressive aphasias (PPA), and to sensorimotor function in Parkinson’s disease (PD) and its atypical variants as well as Huntington’s disease (HD) [1–5]. Semantic variant (sv)PPA and nonfluent agrammatic variant PPA (nfvPPA) are summarized together with bvFTD under the umbrella term frontotemporal dementia (FTD) or – based on a neuropathological diagnosis – frontotemporal lobar degeneration (FTLD). All these neurodegenerative diseases might develop cognitive and behavioral impairment, and, finally dementia in its course.

One of the most intriguing consequences of these alterations is the fact that persons affected by these diseases might develop criminal risk behavior (CB). Such behavior has been shown in studies to occur in FTD [6–8], namely in bvFTD and svPPA, with a much higher frequency than in AD or other syndromes, such as Parkinsonian syndromes (ParkS), HD or vascular dementia (VaD) [9–13]. If persons violate social or legal norms due to changes in behavior, personality and cognition, these incidents may have a substantial impact on the patient’s family and social surroundings and may lead to prosecution. Raising awareness of this problem and characterizing CB in dementia syndromes would help better understanding possible implications of such diseases, identifying possible reasons, and encouraging interdisciplinary endeavors to develop coping strategies. Previous reviews have only provided a broad overview of the prevalence of CB in specific dementia syndromes and mostly focused on antisocial behavior (see for instance, [14–16]).

This review and meta-analysis is the first one systematically and quantitatively investigating and characterizing CB in dementia syndromes. To accomplish this aim, firstly, a systematic literature search was conducted to extract studies that investigated prevalence rates for CB in a broad range of dementia and neurodegenerative syndromes. Secondly, a quantitative meta-analysis aimed at extracting the essential information from the identified studies, i.e., prevalence of CB in the different diseases. We hypothesized higher CB prevalence in FTD, i.e., bvFTD and svPPA, compared to other syndromes. Furthermore, we synthesized the available literature to characterize CB regarding occurrence in disease course, and impact of diversity such as sex / gender. By discussing limitations and open issues of current research, we pave the road for future studies on CB in dementia and to cope with such phenomena in clinical care.

## Methods

### Definition of criminal risk behavior

The definition of CB is inconsistent among literature. Even though the term CB is often used in the literature, no accepted definition has been accepted so far, which is most likely due to different legal systems. In the present review, CB was defined, based on the definition by the Statista Research Department [17], as any behavior that causes harm to other people (or animals), or damages others property, including public property. More specifically, behaviors ranging from harassment, threats, violence, sexual assaults to theft, traffic violations, illegal possession of goods and homicide were considered criminal. Further, behaviors such as public urination and indecent exposure in public were considered as CB, as they violate the law of the region the study was conducted in.

### Pre-registration & rationale

This systematic literature review and meta-analysis was registered at the international prospective register of systematic reviews (PROSPERO). The protocol can be accessed at www.crd.york.ac.uk (registration number CRD42022378824). The meta-analysis was conducted according to criteria of the preferred reporting items for systematic reviews and meta-analyses (PRISMA) statement to guarantee high quality and validity [18].

### Search strategy

The search strategy consisted of several phases. During the first phase, a pilot search at the literature repositories PubMed and Google Scholar (scholar.google.com) was conducted to identify appropriate keywords. The second phase consisted of an extensive systematic search, including title, abstract and full text screening. The electronic database PubMed was searched on 18^th^ October 2022 with the following search strategy: (neurodegeneration OR dementia) AND (criminal OR crime OR criminality OR transgression OR felony OR violation OR offence OR delinquency).

Search results were exported into citation manager Zotero 6.0.17 (zotero.org) and duplicates were removed. If the abstract seemed relevant the full text was assessed and checked for eligibility. Two researchers, LS and MŽ, screened independently titles, abstracts, and full text. Any deviations were discussed until a consensus was reached. An additional systematic search for the term ‘primary progressive aphasia’ was conducted on the 31^st^ of October 2022 to check whether the previous search strategy identified all relevant articles with the search strategy: (primary progressive aphasia) AND (criminal OR crime OR criminality OR transgression OR felony OR violation OR offence OR delinquency). A last search on 5^th^ October 2023 with the search strategy (neurodegeneration OR dementia OR aphasia) AND (criminal OR crime OR criminality OR transgression OR felony OR violation OR offence OR delinquency) revealed one further relevant paper [6]. Another relevant paper [19] was identified via personal communication. The last phase included critical assessment of full text for eligibility.

### Inclusion and exclusion criteria

Studies were considered eligible if fulfilling as inclusion criteria: (i) peer-reviewed empirical case control studies, (ii) reporting CB prevalence in subjects with dementia or neurodegenerative disease, and (iii) applying established diagnostic criteria. Articles of all languages were included if the title and abstract were published in English. Studies, in which the full text was published in another language than English, were assessed by a researcher fluent in the corresponding language. No date or country restrictions were applied. Exclusion criteria were (i) non-reviewed articles, (ii) case studies, (iii) diagnosis not based on recognized diagnostic criteria, and (iv) CB not specific to subjects with dementia.

### Data extraction and synthesis

Data were extracted from the included studies by LS and MLS. When prevalence of CB was not provided in percent, the percentage was calculated based on the numbers supplied. Further, if only prevalence and total number of subjects were given for a dementia subtype, then the number of subjects exhibiting CB was calculated to determine the total prevalence. Results were checked for duplicates, i.e., multiple studies from the same authors, or assessing the same cohort.

For quantitative meta-analysis, (i) mean frequencies of CB were calculated and compared between syndromes with independent samples Kruskal-Wallis tests followed by pairwise comparisons for descriptive purposes. (ii) Re-analysis was performed after normalization of CB prevalence to country-specific overall crime rates to adjust for this potential bias. (iii) Effect sizes (ES), i.e., odds ratios (OR) were calculated for CB in comparison between the several syndromes to control for possible confounding factors. Here, a random effects model was applied, including I^2^ as a measure of heterogeneity, and trim-and-fill analyses. Data were re-analyzed excluding studies deviating in Funnel analysis to secure validity if enough studies were available. For analysis, SPSS version 29 was used, for illustration https://www.mapchart.net/world.html.

### Quality assessment

Potential risk of bias was assessed with the Newcastle-Ottawa scale (NOS) for case-control studies and the Joanna Briggs Institute (JBI) critical appraisal checklist for studies reporting prevalence data (2020) [20–22]. Number of items identified as “yes” were added up. Questions that were not applicable for a study were counted as 0, so results of the quality assessment should be assessed carefully. LS checked for risk of bias.

### Statement on diversity, equity & inclusion (DEI)

Institutions involved in the study support diversity and consider it as a prerequisite for excellent science. The authors aimed at including diversity in the analyses, which is reflected in the unbiased systematic literature search approach including data worldwide, i.e., from different ethnicities, as far as available. Furthermore, sex/gender was included as variable of interest and articles screened, accordingly, for this information. Further prospective studies are suggested including more diverse populations as a desideratum for the future. An international prospective study on CB in dementia is already scheduled in the framework of the Neuropsychiatric International Consortium on Frontotemporal Dementia (NIC-FTD).

### Patient & public involvement (PPI)

Results have been discussed with subjects suffering from dementia and relatives/caregivers, and will be published in the media.

## Results

### Characteristics of studies identified by systematic review

**Fig. 1:**
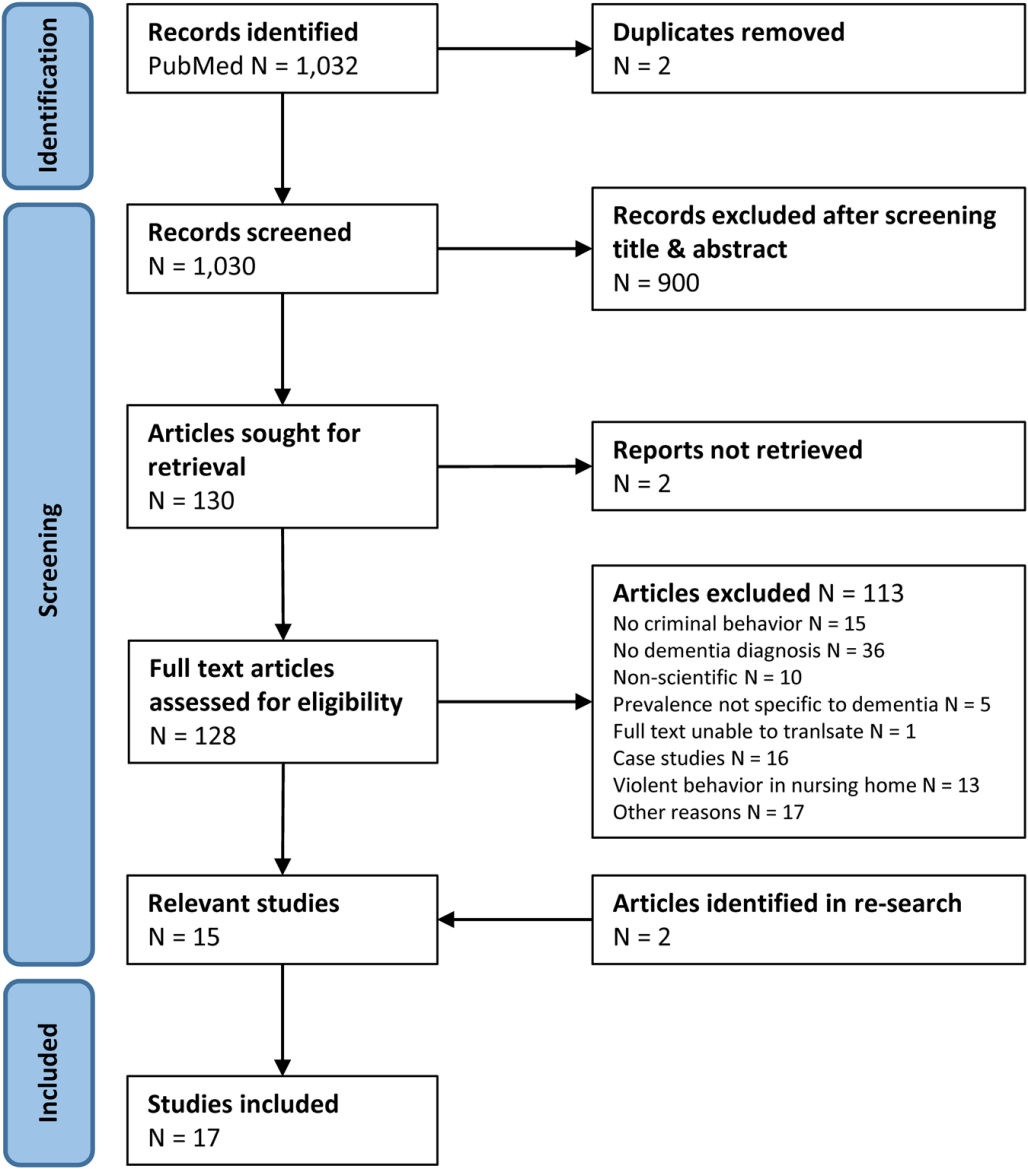
Flow of information through the different phases of the systematic review investigating criminal risk behavior in dementia and neurodegenerative disease according to the preferred reporting items for systematic reviews and meta-analyses (PRISMA) statement [18].

Figure 1 illustrates the flow of the systematic literature search. Finally, 17 relevant studies were identified that investigated CB prevalence in dementia and neurodegenerative syndromes based on the operationalization of CB in the methods section. Those behaviors included, in the several studies, a wide range from indecent behaviors such as sexual advances, harassment, exposing body and urinating in public, behavior causing harm to other people or animals, physical aggression, damaging others property, housebreaking, trespassing, theft, refusing to pay, fare evasion, illegal financial activity, traffic violation, speeding, hit and run accidents, driving under the influence of alcohol, alcohol and substance abuse, to behavior resulting in police interaction. Supplemental Table S1 describes details for the studies.

**Fig. 2:**
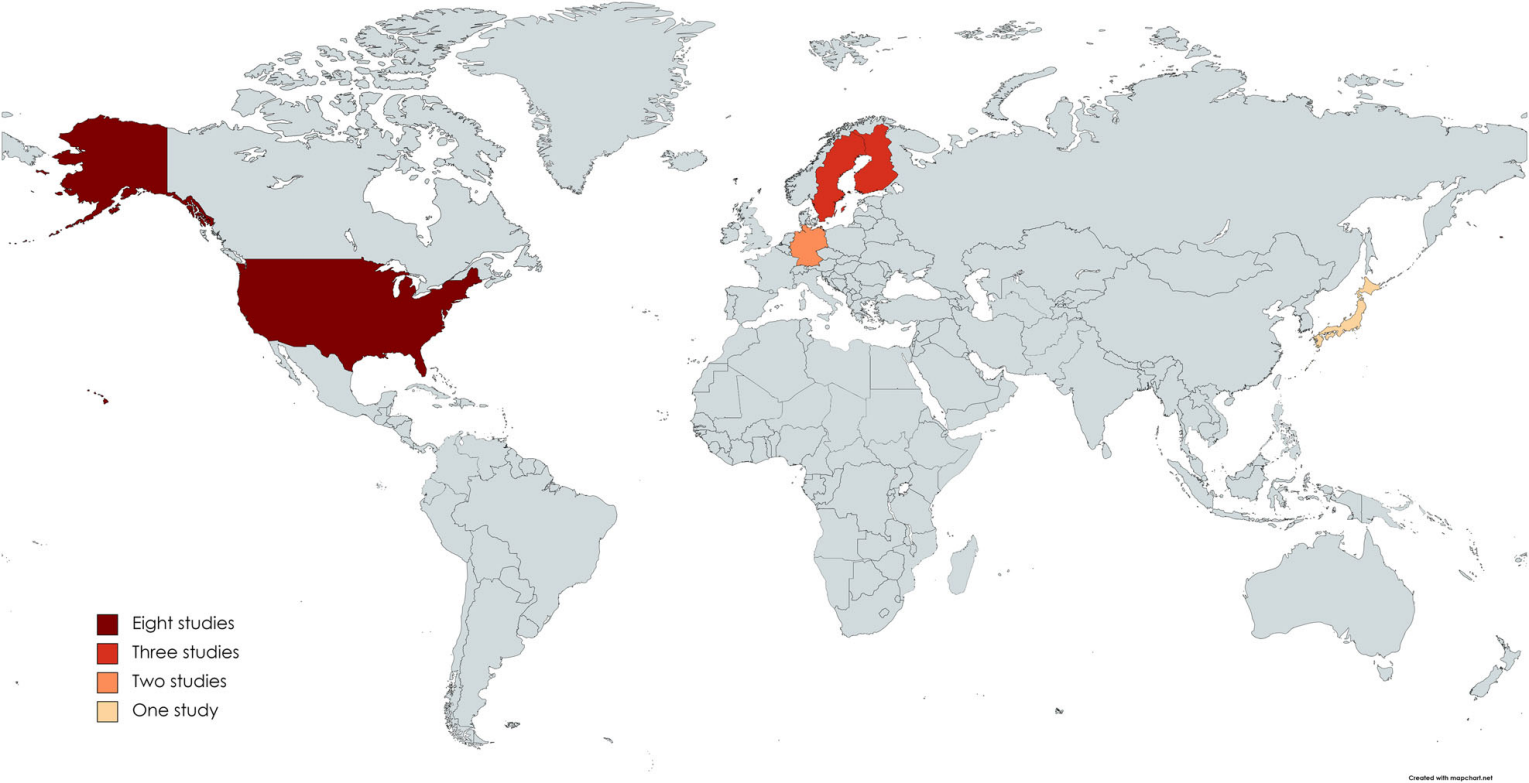
Number of studies investigating criminal risk behavior in dementia and neurodegenerative syndromes per country worldwide. Numbers of studies are color-coded for each country.

**Fig. 3:**
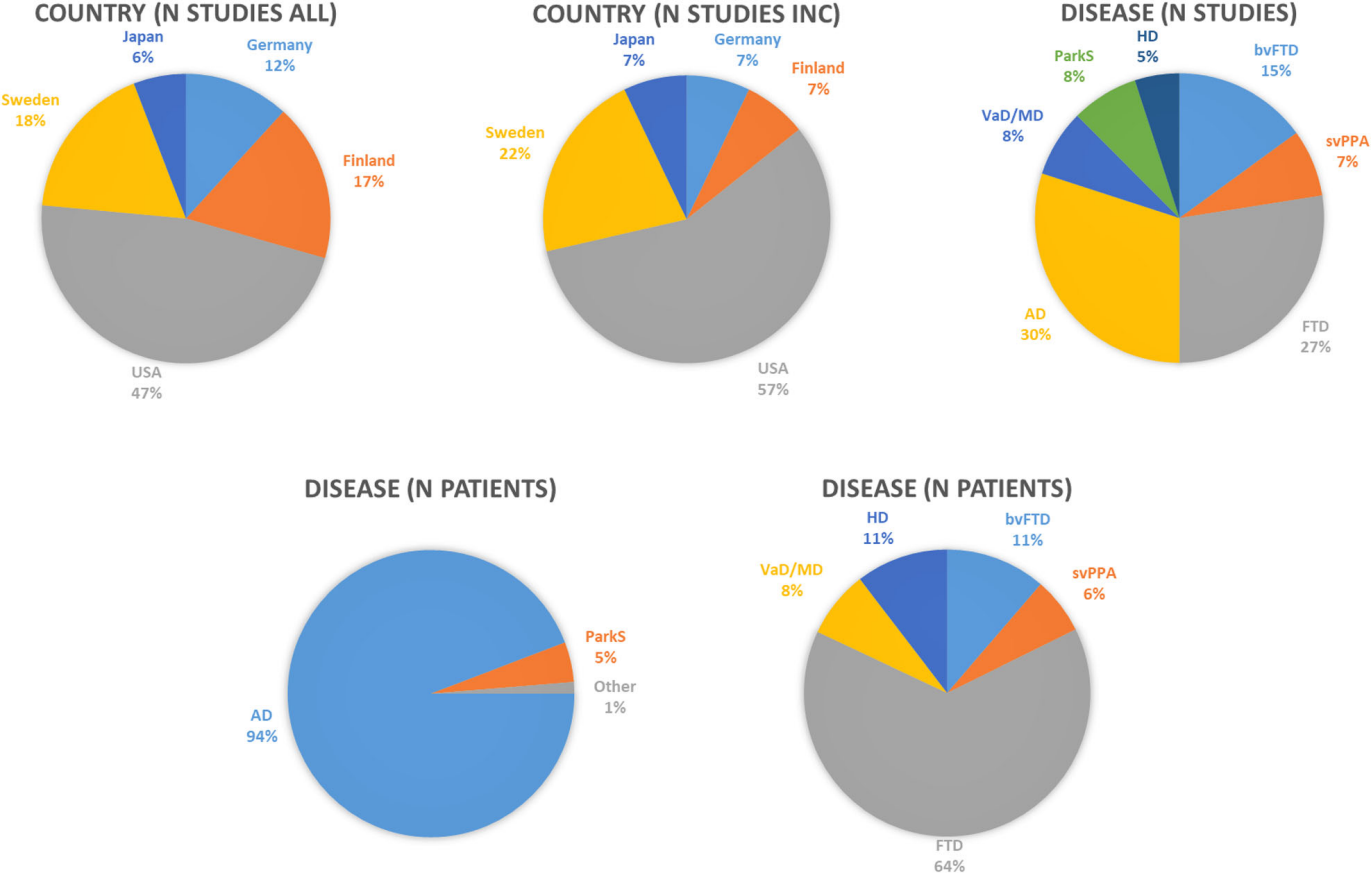
Information for studies regarding country of origin, neurodegenerative diseases and numbers of subjects included. *Upper left:* Percentage of country of origin for all identified relevant studies before exclusion of overlapping cohorts. *Upper middle:* Percentage of country of origin for studies after exclusion of overlapping cohorts. *Upper right:* Percentage of number of studies involving several disease cohorts included in quantitative meta-analysis, i.e., after exclusion of overlapping cohorts. *Lower panels:* Percentage of total number of patients per diagnostic group included in quantitative meta-analysis, i.e., after exclusion of overlapping cohorts. *Left:* Numbers for largest cohorts, i.e., Alzheimer’s disease (AD) and Parkinsonian syndromes (ParkS) and other groups. *Right:* Subanalysis for other groups (corresponding to 100%), i.e., frontotemporal dementia (FTD), behavioral variant (bv)FTD, Huntington’s disease (HD), semantic variant primary progressive aphasia (svPPA) and vascular dementia/mixed dementia (VaD/MD).

As shown in Figs. 2 & 3, upper left panel, studies originated from different countries, with largest contributing country being the U.S.A., followed by Scandinavian countries, i.e., Sweden and Finland, and additionally Germany and Japan. Accordingly, the U.S.A. and European countries were overrepresented, whereas Asia, Africa, and Australia were underrepresented, beside the numerous other American and European countries, which might be regarded as a geographical bias.

### Characteristics of studies identified by meta-analysis after exclusion of duplicates

We checked the 17 identified studies for including overlapping cohorts to prevent a duplication bias. Diehl-Schmid et al. [9] investigated CB in FTD and AD. A former article by the same group [23] reported results of a subsample with exactly the same questionnaire. Accordingly, the latter article (in German language) was excluded from the meta-analysis to prevent a duplication bias due to overlapping cohorts. In the result, our systematic meta-analysis contained articles in English only.

Talaslahti et al. conducted two nationwide register studies in Finland investigating CB in one [8] or four years [7] preceding the diagnosis of neurocognitive disorder in a very large cohort. Because both studies involved the same cohort and our meta-analysis aimed at general frequency of CB, we included the study covering a range of four years [7] and excluded the study covering one year only [8]. Ginters et al. [6] investigated CB after the diagnosis of dementia in the Nationwide Finnish Register covering the same cohort. Because of the overlap with the cohort in Talaslahti et al. [7], we excluded this study, particularly given the fact that all three studies were based on police registry and a substantial decline in police contact had been reported after diagnosis there.

Several papers by Liljegren et al. reported CB frequency in a larger histopathologically characterized Swedish cohort with dementia. The most recent and largest study [24] investigated a broad range of criminal and socially inappropriate behavior. Although another study by the same group [25] was based on a smaller subsample of this cohort, it was focused on physical aggression toward other persons or living creatures only, whereas exactly this kind of CB was not included in [24]. Accordingly, we decided to include both studies, because they investigated the same cohort but different kinds of CB. Another study [11] investigated police interactions in dementia in a cohort overlapping with the larger studies [24, 25]. However, as this study focused on CB that led to police interaction only, we still included their findings as assessing CB with a different measure, i.e., based on police interaction. Moreover, aggression and other kinds of CB were included in this study.

Accordingly, we excluded three studies [6, 8, 23] from the systematic meta-analysis to avoid a bias of overlapping cohorts to prevent a duplication bias.

Figure 3 and Supplemental Table S2 describe the main characteristics of the remaining 14 studies. The distribution of countries contributing studies remained with dominance of the U.S.A., followed by Scandinavian countries, Germany and Japan (Fig. 3, upper middle panel).

If one regards the number of studies investigating one disease, most studies investigated AD (12) and FTD (11), followed by bvFTD (6), svPPA (3), VaD / mixed dementia (VaD/MD) (3), ParkS (in sum three studies, with one study for progressive supranuclear palsy, corticobasal syndrome, and Lewy body dementia / PD dementia, LBD/PDD, respectively), and HD (2). Relative distribution is illustrated in Fig. 3, upper right panel. Obviously, studies on AD and FTD dominated.

In total, studies included overall a very large number of 236,360 persons with dementia/neurodegenerative syndromes (Table S2). Note that for calculation of total number of patients, numbers for FTD if calculated from bvFTD/svPPA were not included. As illustrated in Fig. 3, left lower panel, 94% of the cohort represented persons with AD (223,169), and 5% persons with several ParkS (10,727), mainly driven by two comprehensive U.S. American [26] and Finnish [7] studies. However, the remaining other studies (see Fig. 3, right lower panel), still contained considerable numbers of persons, i.e., 1,925 for FTD, 339 for bvFTD, 191 for svPPA, 227 for VaD/MD, and 312 for HD. One can conclude that the systematic literature search revealed sufficient numbers of studies and subjects for a quantitative meta-analysis, although a few diseases, namely the most frequent dementia syndrome AD, were overrepresented. Note that we controlled for such a bias in the following analyses.

### Results of quantitative meta-analysis

For the quantitative meta-analysis, firstly, we calculated and compared mean frequencies of CB for each syndrome, and, secondly, calculated ORs for CB in comparison between several syndromes. Whereas the first analysis was conducted to provide a general description, the second analysis enabled a much better controlled quantitative meta-analysis. For the first analysis, we recalculated statistics after normalization of prevalence for CB to country-specific overall crime rates to adjust for this potential bias.

Note that we conducted the quantitative meta-analyses without differentiating between the different CBs, because the number of studies investigating specific CBs was too low for respective sub-meta-analyses.

### Mean frequency of criminal risk behavior in dementia and neurodegenerative syndromes

**Fig. 4:**
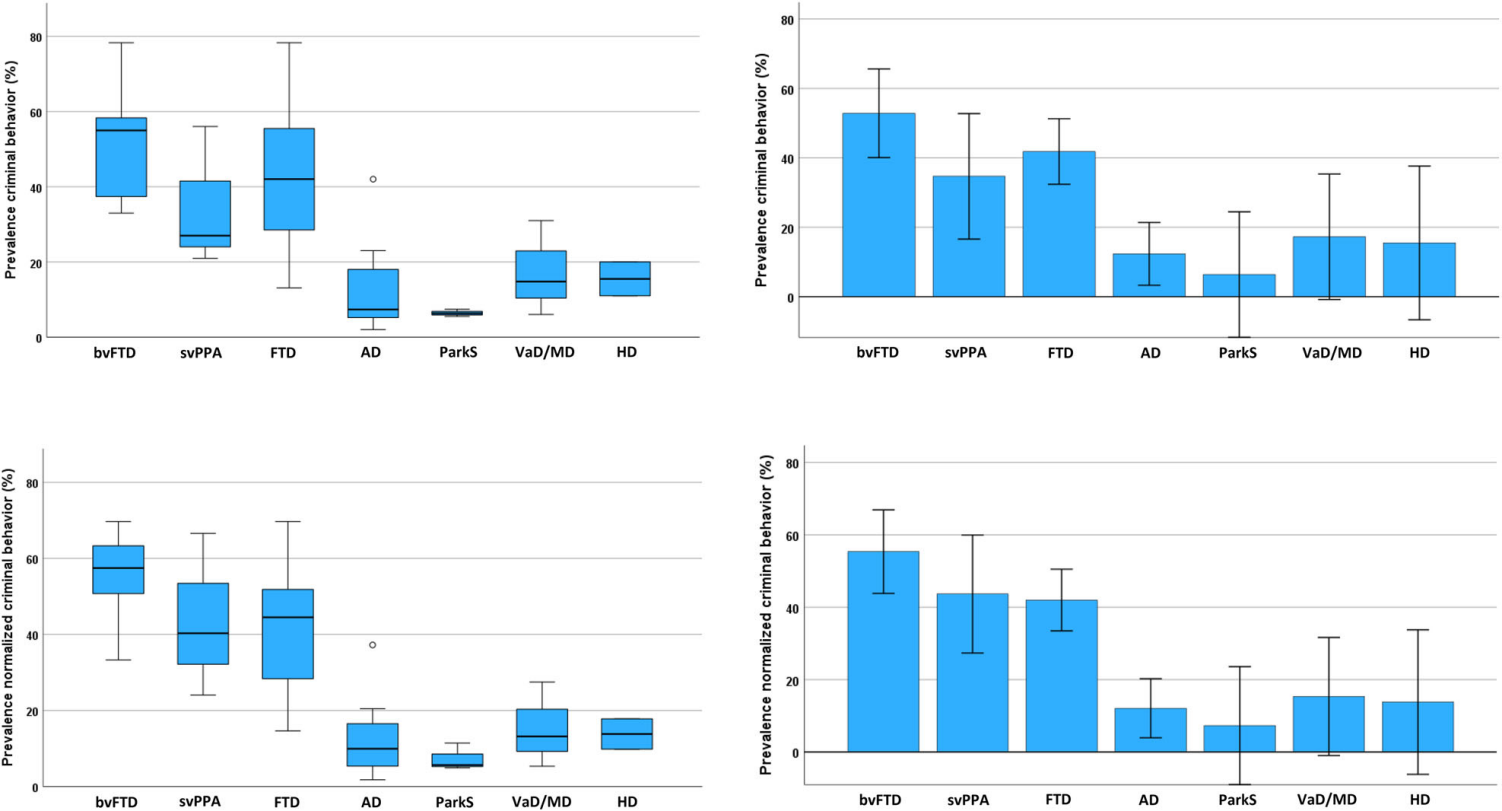
Mean prevalence of criminal risk behavior in dementia and neurodegenerative syndromes. *Upper panel:* Absolute values. *Lower panel:* Values normalized to mean country-specific crime rates. Box plots (left) and bar charts representing mean ± standard deviation (right). For statistical comparisons see Fig. 5. Abbreviations: AD Alzheimer’s disease, bvFTD behavioral variant FTD, FTD frontotemporal dementia, HD Huntington’s disease, ParkS Parkinsonian syndromes, svPPA semantic variant primary progressive aphasia, VaD/MD vascular dementia/mixed dementia.

For our first quantitative meta-analysis, we calculated mean CB prevalence for each cohort, and compared values between the several disease groups. Figure 4 (and Table S2) shows mean CB prevalence in dementia and neurodegenerative syndromes, upper panels with absolute values, lower panels with values normalized to mean country-specific crime rates. Both, box plots and bar charts are shown.

On a descriptive level, absolute CB prevalence was highest in bvFTD (52.8 ± 16.3%; mean ± standard deviation), followed by svPPA (34.7 ± 18.7%), with values for FTD (41.8 ± 19.8%) between bvFTD and svPPA as expected. Much lower mean CB prevalence was observed in the other syndromes, i.e., AD (12.3 ± 11.6%), VaD/MD (17.3 ± 12.7%), HD (15.5 ± 6.4%), and especially ParkS (6.4 ± 1.0%). For a quantitative comparison, we applied an independent samples Kruskal-Wallis test followed by pairwise comparisons. We included only disease groups with at least three available studies in this statistical analysis. Accordingly, HD was excluded, because only two studies were available.

**Fig. 5:**
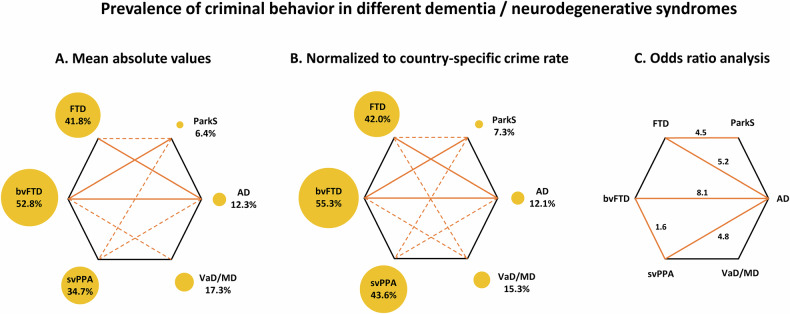
Results for statistical comparisons of prevalence of criminal risk behavior in several dementia and neurodegenerative syndromes. *Left and middle panel*: Independent samples Kruskal-Wallis test followed by pairwise comparisons. Orange lines illustrate significant comparisons (p < 0.05, one-sided test), dashed or solid if surviving Bonferroni correction for multiple tests. Absolute values (**A**; left panel) and values after normalization to mean country-specific crime rates (**B**; middle panel). Size of circles corresponds to mean frequencies of criminal risk behavior. *Right panel* (**C**): Orange lines illustrate significant results for group comparisons of odds ratio analysis. Odds ratios are reported for significant comparisons (p < 0.05). Note that all statistical analyses were conducted only, if at least three studies were available for each group. *Abbreviations*: AD Alzheimer’s disease, bvFTD behavioral variant FTD, FTD frontotemporal dementia, HD Huntington’s disease, ParkS Parkinsonian syndromes, svPPA semantic variant primary progressive aphasia, VaD/MD vascular dementia/mixed dementia.

The Kruskal-Wallis test revealed significant differences of CB prevalence between the groups (p < 0.001, two-sided). Figure 5, left panel, shows statistical results for post hoc pairwise comparisons (left panel; p < 0.05, one-sided). Significant comparisons are illustrated with dashed lines, solid lines if surviving Bonferroni correction for multiple comparisons. Prevalence of CB was significantly higher in bvFTD than AD and ParkS, and higher in FTD than AD even after Bonferroni correction. Further significant differences were found for bvFTD vs. VaD/MD, svPPA vs. AD and ParkS, and FTD vs. ParkS.

### Mean frequency of criminal risk behavior in dementia and neurodegenerative syndromes after normalization to country-specific crime rates

It is well known that crime rates differ between countries, where high poverty levels and unemployment may inflate country’s crime rate, whereas strict police enforcement and severe sentences might reduce crime rates. This factor can be regarded as a potential bias (see different crime rates for the same syndrome in different countries in Table S1). To adjust for these differences we normalized CB prevalence in the different syndromes to country-specific overall crime rates per study. Country-specific overall crime rates were extracted from https://worldpopulationreview.com/country-rankings/crime-rate-by-country on 2^nd^ of January 2023 (Table S2). Country-specific crime rate is calculated by dividing the total number of reported crimes of any kind by the total population, then multiplying the result by 100,000 to receive crime rate as X number of crimes per 100,000 people. Normalized, i.e. country-adjusted, CB frequency was calculated for each disease group in each study with the following formula: ssCB / csCR * mean csCR, i.e., study-specific CB prevalence / country-specific crime rate x mean-country specific crime rate for all groups.

Figure 4, lower panels, shows mean normalized CB prevalence in the different syndromes. Prevalence of CB was again highest in bvFTD (55.3 ± 13.0%), followed by svPPA (43.6 ± 21.5%) and FTD (42.0 ± 17.8%). Much lower mean CB prevalence was again observed in AD (12.1 ± 10.0%), VaD/MDD (15.3 ± 11.2%), HD (13.8 ± 5.7%), and in particular ParkS (7.3 ± 3.6%). Values were compared with an independent samples Kruskal-Wallis test followed by pairwise comparisons. Again, HD was excluded, because only two studies were available.

The Kruskal-Wallis test revealed significant differences of normalized CB prevalence between the groups (p < 0.001, two-sided). Figure 5, middle panel, shows statistical results for post hoc pairwise comparisons (p < 0.05, one-sided). Significant comparisons are illustrated with dashed lines, solid lines if surviving Bonferroni correction for multiple comparisons. Prevalence of CB was higher in bvFTD than AD and ParkS, and higher in FTD than AD even after Bonferroni correction. Further significant differences were found for bvFTD vs. VaD/MD, svPPA vs. AD and ParkS, and FTD vs. ParkS and VaD/MD.

### Odds ratios for criminal risk behavior in comparison between dementia and neurodegenerative syndromes

**Fig. 6:**
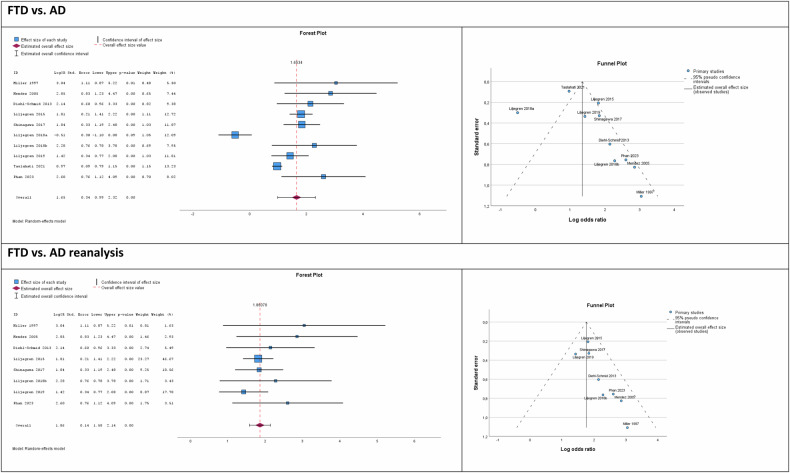
Results for the odds ratio (OR) analysis of criminal risk behavior prevalence in frontotemporal dementia (FTD) vs. Alzheimer’s disease (AD; other comparisons see Figure S1). Forest plots show higher prevalence in FTD than AD with an effect size of 1.653, corresponding to an OR of 5.223; the reanalysis without Liljegren et al. [25] and Talaslahti et al. [7], studies that deviated in the Funnel analysis, showed an ES of 1.861, corresponding to an OR of 6.430.

For the last part of the quantitative meta-analysis, we compared CB prevalence between the several dementia syndromes by calculating ORs. Here, we included only pairs of dementia syndromes where at least three studies were available for each syndrome. Results are summarized in Fig. 5, right panel, and reported in detail in Fig. 6 and Figure S1 in the Supplement. Note that this analysis was based on studies using the same methodological approach for the respective syndromes. Accordingly, numbers of studies included were smaller than in the aforementioned approaches, but, most crucially, this approach compared CB prevalence in different syndromes measured with exactly the same approach, and corrected for different cohort sizes.

As illustrated in Figs. 5 and 6 (Forest and Funnel plots), studies for FTD showed higher prevalence rates for CB than those for AD. Effect size was 1.653, corresponding to an OR of 5.223, p < 0.001 (two-tailed), I^2^ = 90.2%. A trim-and-fill analysis based on observed and imputed values confirmed this finding (ES = 1.348; p < 0.001). A re-analysis without Liljegren et al. [25] and Taslahati et al. [7], studies that deviated in the Funnel analysis, showed an ES of 1.861, corresponding to an OR of 6.430, p < 0.001, I^2^ = 0%.

For the following analyses Forest and Funnel plots are illustrated in Figure S1 (summary again in Fig. 5). The analysis comparing the FTD subtypes, i.e., bvFTD and svPPA, to AD, revealed higher CB prevalence in bvFTD (ES = 2.089, OR = 8.077, p < 0.001, I^2^ = 0%, trim-and-fill analysis ES = 1.984, p < 0.001) and svPPA (ES = 1.574, OR = 4.826, p < 0.001, I^2^ = 0%, trim-and-fill analysis ES = 1.574, p < 0.001) than AD. Moreover, CB prevalence was higher in bvFTD than svPPA (ES = 0.443, OR = 1.557, p = 0.035, I^2^ = 0%, trim-and-fill analysis ES = 0.443, p = 0.035).

Although FTD showed higher CB prevalence than VaD/MD (ES = 0.657, OR = 1.929, I^2^ = 68.1%), this effect was not significant (p = 0.114). Prevalence of CB was also comparable between AD and VaD/MD (ES = −0.323, OR = 0.724, I^2^ = 70.4%, p = 0.512). For ParkS, CB prevalence was higher in FTD (ES = 1.499, OR = 4.477, I^2^ = 82.3%, p = 0.005, trim-and-fill analysis ES = 1.499, p = 0.005), but not AD (ES = −0.147, OR = 0.863, I^2^ = 34.4%, p = 0.531).

## Discussion

Our study aimed at investigating CB prevalence in dementia and neurodegenerative syndromes. The systematic literature search identified 17 relevant studies that investigated the frequency of CB in those syndromes. After exclusion of duplicates, 14 studies remained for the systematic and quantitative meta-analysis. As far as we know, this is the first systematic literature review and meta-analysis investigating CB prevalence in dementia. Previous reviews provided an overview of CB prevalence in FTD and have discussed the potential implications for criminality in FTD [14, 16], but did not investigate those issues systematically and in a wider perspective across all dementia syndromes.

### Prevalence of criminal risk behavior is highest in bvFTD, followed by svPPA, but rather low in AD, vascular dementia, Huntington’s disease and lowest in Parkinsonian syndromes

In total, studies included overall a very large number of 236,360 persons with dementia and neurodegenerative syndromes. Although AD was overrepresented, sufficient numbers of persons with other syndromes were included, ranging from movement disorders, i.e., ParkS and HD, to other diseases affecting behavior, cognition, and language function, namely bvFTD, svPPA, and vascular dementia.

A wide range of methods was applied in the studies to investigate CB. Some studies focused on specific kinds of CB, such as driving under influence, or physical aggression, whereas others used rather broad approaches covering CB in its full extent. Some studies were conducted in rather small clinical cohorts, some were done in large population-based studies for instance by referring to police registries. Moreover, countries of origin varied with dominance of the U.S.A. and Europe, whereas Asia, Africa, Australia and the other American countries were under-represented. Remarkably, crime rates differ between countries. For instance, relatively low CB prevalence was reported by studies from Japan [13] and Finland [6–8], corresponding with lowest country-specific general crime rates in these countries (see Tables S1 and S2).

Despite these limitations, all studies used established international diagnostic criteria or even neuropathological disease confirmation guaranteeing a correct identification of diagnosis. To minimize influences of the aforementioned potential biases, we controlled for different cohort sizes and different methods in estimating CB prevalence and country-specific crime rates in the following analyses. For the quantitative meta-analysis, we applied several analysis methods to cross-validate findings. We calculated mean CB prevalence for each syndrome, recalculated statistics after normalization of CB prevalence to country-specific overall crime rates to adjust for this potential bias, and calculated odds ratios for CB in comparison between the several syndromes.

All quantitative analyses revealed a very consistent picture (see Fig. 5). *Prevalence of criminal risk behavior was highest in bvFTD, followed by svPPA (accordingly also in FTD), but rather low in AD, vascular dementia, HD and lowest in ParkS*. Levels reached more than 50% in bvFTD and about 40% in svPPA, but even approximately 15% in vascular dementia and HD, 10% in AD and less than 10% in ParkS.

One can conclude that CB has to be regarded as a frequent syndrome in dementia and neurodegenerative syndromes worldwide. One might even hypothesize that – as most studies investigated CB retrospectively – it is likely that numbers are underestimated. If CB is observed in the elderly for the first time in their life such diseases have to be taken into account and persons thoroughly investigated by clinicians. Moreover, close interdisciplinary cooperation between medical and legal systems is required to develop coping strategies.

### Large prospective international studies are warranted applying homogeneous methods in assessing criminal risk behavior in dementia syndromes

Although our systematic review and meta-analysis yields a comprehensive view on the overall CB prevalence in dementia syndromes, it has limitations. Generally one has to admit – as already discussed in the introduction section – that there is no generally accepted definition of CB and that, accordingly, studies show high variability. Whereas some studies screened across a wide range of CB, a few studies focused on single kinds of CB only. Our meta-analysis could not investigate the several kinds of CB in sub-meta-analyses due to low numbers of studies for specific CBs. Moreover, CB was analyzed for different time periods, where for instance [7, 8] screened for CB in time windows before the diagnosis of disease, [6] in the same cohort after diagnosis, others across the whole time of disease.

Information was also based on different sources, ranging from police registries to medical records. For some studies, only limited information was available, such as for [27]. Criminal risk behavior was only reported for right-sided bvFTD, but not for left-sided bvFTD. Although several studies suggested different CB patterns in different syndromes, presumably related to their specific behavioral and cognitive impairments, information was not systematically available and could not be meta-analyzed. As already mentioned, available studies were not balanced for country and cohort size and included much more persons with common diseases, i.e., AD, in contrast to rare or orphan diseases.

Although neural correlates of CB have been investigated, findings have to be replicated in larger cohorts. Antisocial behavior/CB has been associated with right hemisphere in bvFTD [12, 27], and bilateral frontomedian atrophy in a broader dementia group [19], – regions well known to be crucially involved in bvFTD and related to sociocognitive and moral functions [28–34], – or Transactive response DNA binding protein of 43 kDa (TDP-43)/non-tau as the predominant pathology in FTD/FTLD [11, 24].

In conclusion, *large prospective international studies are warranted, applying homogeneous methods, i.e., standardized questionnaires with strict definition of CB, to assess CB in a wide range of dementia syndromes systematically*.

### Criminal risk behavior is more frequent than in the general population in early disease course, i.e., before diagnosis, but declines thereafter below population levels

In the following, results of the systematic literature review are discussed, although quantitative meta-analyses could not be conducted here due to limitations of statistical power (not enough studies available). Criminal risk behavior in FTD is most likely caused by the neurodegenerative disease itself. Most of the patients showed CB for the first time in their life and had no previous records of criminal activity [9, 24]. A very large nationwide Finnish register study [7] cross-referenced medical reports with records from the police register in approximately 90,000 subjects. It demonstrated that CB may manifest already four years before an official diagnosis of FTD, with lower prevalence also in AD and LBD/PDD. Authors of the study compared CB to the whole population of Finland. Criminal risk behavior in subjects with FTD and AD was significantly more frequent than in general population, but not in LBD/PDD. Ginters et al. [6] investigated CB in the same Finish cohort, but after diagnosis. CB prevalence was reduced by up to 50%. CB was not higher, rather lower, after diagnosis if compared with the general Finnish population. More specifically, number of crimes was comparable for FTD in women, but lower for the other cohorts. The number of crimes committed by patients with FTD seemed to decrease with age [7]. Shinagawa et al. [13] supported this finding by demonstrating a decrease in CB after initial consultation in bvFTD, svPPA and AD. However, the situation might be different for specific CB, where [35] did not find differences in car crashes between AD and the general population, neither before nor after diagnosis.

One may conclude that *CB prevalence seems to be more frequent in FTD and AD in early disease course than in the general population before diagnosis, presumably in prestages such as mild behavioral and/or cognitive impairment, but declines thereafter finally leading to lower prevalence in dementia after diagnosis if compared with the general population*.

### Criminal risk behavior is more frequent in men than women in dementia

Another noteworthy differentiation of CB in dementia is related to diversity, here sex/gender. More men than women committed crimes in the year or the four years before diagnosis in FTD, AD and LBD/PDD [7, 8]. After diagnosis, men showed four times more CB than women in FTD, seven times more in AD and 2.3 times more in LBD/PDD [6]. Shinagawa et al. [13] showed that men were overrepresented in their FTD and AD cohort with CB before first consultation. Liljegren et al. [10] reported that men were more likely than women to make sexual advances, all patients who urinated in public were men. Further studies underline the male overrepresentation for CB in neurodegenerative disease, here for arrests due to driving under the influence of alcohol in AD with men showing six to eight times higher risk than women [26], for CB in HD [36], or physical aggression in FTLD, AD and VaD/MD [25].

In sum, *men seem to be generally overrepresented compared to women in showing CB due to dementia or neurodegenerative disease*. Future studies shall assess impacts of diversity, such as sex/gender, ethnicity or social factors, on criminal activity.

### Early diagnosis of disease is of uttermost importance to prevent stigmatization due to criminal risk behavior

Finally, one has to prevent a further stigmatization of persons with dementia. Of note, most offences committed were minor such as indecent behavior, traffic violations, theft, damage to property, but also physical violence or aggression occurred (see Table S1). Crime rates are lower than in the general population after diagnosis [6], after higher values before diagnosis [7]. Note, moreover, that overall CB in dementia is rather low as subtypes of FTD are orphan diseases in contrast to frequent AD.

Hence, *sensitivity for CB as possible early signs of dementia and earliest diagnosis and treatment are of uttermost importance*. Beside early diagnosis and treatment of persons affected one has to discuss *adaptations of the legal system*, such as increasing awareness of offences due to those diseases, and taking into account diseases in respective penalties and in jails.

### Conclusion

In conclusion, our systematic review and quantitative meta-analyses show that criminal risk behavior is frequently observed in dementia and neurodegenerative syndromes, in particular in the subtypes of frontotemporal dementia, i.e., behavioral variant frontotemporal dementia and semantic variant primary progressive aphasia, a consistent finding across several countries and cultures. Prevalence of criminal risk behavior seems to be more frequent in early disease course of dementia than in the general population, i.e., before diagnosis, but declines thereafter. Men seem to be generally overrepresented compared to women. Accordingly, criminal risk behavior committed for the first time at mid-age could be an indicator of dementia and particular frontotemporal dementia and shall lead to early diagnosis and treatment. As present studies show a wide variability in assessment methods and cohorts investigated, and had been conducted in a minority of countries world-wide, large prospective international studies are warranted that systematically apply homogeneous methods and standardized questionnaires in assessing criminal risk behavior in different dementia syndromes.

## Supplementary information

Supplemental Material
