# Supplementary material for: Criminal minds in dementia: A systematic review and quantitative meta-analysis
Source: Transl Psychiatry. 2025 Aug 28;15:324. doi: 10.1038/s41398-025-03523-z (PMC12394586; doi:10.1038/s41398-025-03523-z)
Supplement: Supplementary file 1 — Supplemental Material [file 41398_2025_3523_MOESM1_ESM.docx]

Table S1 Relevant studies identified by systematic literature review

| **Study info** |  | **Demographics** |  | **Population** | |  |  |  |  | **Criminal behavior** | |  |  | | **Quality score** |
| --- | --- | --- | --- | --- | --- | --- | --- | --- | --- | --- | --- | --- | --- | --- | --- |
| **Author (year)** | **Design** | **Country, Setting,**  **Time period** | **N**  **male:female**  **age (years)** | **Diagnosis**  **(Criteria)** | **Assessment**  **Dementia; Criminality** | | **Control** | **Disease duration**  **(years)** | **Dementia severity** | **Prevalence**  **N with CB/total N (%)** | **Most prevalent types of crimes** | | | **Summary of findings** | **JBI & NOS** |
| Diehl et al. (2006) | R | Germany, Cognitive disorder center, Technical University Munich,  Structured caregiver interview,  1998-2004 | N=74  48:26  56.6 (bvFTD)  57.8 (svPPA)  66.8 (AD) | bvFTD  svPPA  (Neary et al. 1998);  AD  (ICD-10) | MMSE;  Standardized interview of caregivers | | None | 6.1 (bvFTD)  8.5 (svPPA)  5 (AD) | NS | FTD 22/41 (53.7)  bvFTD 15/30 (50)  svPPA 7/11 (63,6)  AD 1/33 (3) (0.03) | bvFTD theft & assault > housebreaking > body exposed or urinating in public > damage to someone else's property; svPPA theft > assault & damage to someone else's property > body exposed or urinating in public > housebreaking; AD housebreaking | | | CB more frequent in FTD, bvFTD and svPPA than AD;  No significant differences between women and men | JBI 7/9  NOS 5/9 |
| Diehl-Schmid et al. (2013) | R | Germany,  Cognitive disorder center, Technical University Munich,  Structured caregiver interview | N=83  51:32  55.6 (bvFTD)  58.8 (svPPA)  66.8 (AD) | bvFTD  svPPA  (Neary et al. 1998);  AD  (ICD-10) | Standardized interview of caregivers | | None | 5.7 (bvFTD)  7.3 (svPPA)  4.9 (AD) | NS | FTD 27/50 (54)  bvFTD 17/32 (53)  svPPA 10/18 (56)  AD 4/33 (12) | bvFTD theft > indecent behavior > assault & housebreaking > willful damage to property; svPPA theft & indecent behavior > willful damage to property > assault > housebreaking; AD indecent behavior > assault > housebreaking | | | Patients with CB had twice the disease duration of patients without CB;  Men and women had similar rates of CB; no differences of age or education between patients with or without CB | JBI 8/9  NOS 4/9 |
| Ginters et al. (2023) | R | Finland,  Finnish Care Register for Health Care & Finnish National Police Register,  1998-2015 | N=92,189  33,294:58,895 | FTD  AD  LBD/PDD  (ICD-10) | ICD-10;  Police register | | Finnish population (age-matched) | NS | NS | At least one crime after diagnosis:  FTD 44/1059 (4.2)  AD 968/80,540 (1.2)  LBD/PDD 366/10,590 (3.5)  Cumulative incidence across 10 years after diagnosis:  FTD NS/NS (male 24.6/female 2.1)  AD NS/NS (5.7/0.7)  LBD/PDD NS/NS (12.6/4.6) | Traffic offences > property > violence > other > alcohol > sexual | | | FTD and LBD/PDD with higher crime rate than AD;  Higher crime rates in men than women;  Number of crimes compared with general population comparable for FTD in women, but lower for other cohorts;  After diagnosis, CB decreases by up to 50% (see comparison with Talaslahti et al. 2021 & 2023) | JBI 8/9  NOS 6/9 |
| Liljegren et al. (2015) | R | USA, University of California, San Francisco, Memory and  Aging Center,  Medical records,  1999-2012 | N=2397 | bvFTD  svPPA  PSP  CBS  AD  HD  VaD  MCI  (established criteria; partly, N=31, histopathology) | MMSE, CDR;  Medical records | | None | NS | MMSE (mean):  19 (bvFTD) 17 (svPPA)  19 (AD) | bvFTD 64/171 (37.4)  svPPA 24/89 (27.0)  PSP 4/63 (6.3)  CBS 4/73 (5.5)  AD 42/545 (7.7)  HD 6/30 (20.0)  VaD 9/61 (14.8)  MCI 8/243 (3.3) | (five most prevalent reported)  bvFTD theft > traffic violations > sexual advances > violence > trespassing;  svPPA theft > traffic violation > violence > trespassing, sexual advances, insubordination & speeding;  AD traffic violation > violence > theft, insubordination & hit and run | | | bvFTD shows more frequent CB in all categories except hit-and-run accidents;  men were more likely than women to make sexual advances; all patients who urinated in public were men | JBI 7/9  NOS 4/9 |
| Liljegren et al. (2018a) | R | Sweden,  Medical records,  1967-2013 | N=281  124:157  75 | FTD/FTLD  AD  VaD/MD  (Histo-pathology) | Neuro-pathological confirmation;  Patients medical notes | | AD  VaD/MD | 8 | NS | FTD/FTLD 29/97 (30)  AD 42/101 (42)  VaD/MD 26/83 (31) | Only physical aggression exerted to harm  another living person or creature investigated:  Victims health staff > other patients, but also family members, unknown people and animals | | | Patients with FTD/FTLD show physical aggression earlier in disease course and in a higher frequency than those with AD;  Men overall more physically aggressive than women | JBI 7/9  NOS 6/9 |
| Liljegren et al. (2018b) | R | Sweden,  Medical records  1967-2013 | N=281  124:157  75 | FTD/FTLD  AD  VaD/MD  (Histo-pathology) | Neuro-pathological confirmation;  Patients medical notes | | AD  VaD/MD | 8 | NS | FTD/FTLD 16/97 (16.5)  AD 2/101 (2.0)  VaD/MD 5/83 (6.0) | CB resulting in police interaction: FTD/FTLD aggression > traffic incidents > shoplifting & refusing to pay;  AD aggression & traffic incidents;  VaD/MD aggression > refusing to pay | | | Police interactions due to CB of patients with FTD/FTLD tended to be more often than for AD or VaD/AD;  TDP-43 was the predominant pathology in FTD/FTLD patients that interacted with the police | JBI 7/9  NOS 6/9 |
| Liljegren et al. (2019) | R | Sweden,  Medical records,  1967-2017 | N=220  92:128  FTD/FTLD 70  AD 76 | FTD/FTLD  AD  (Histo-pathology) | Neuro-pathological  confirmation;  Patients medical notes | | AD | 8 (FTD/FTLD)  10 (AD) | NS | FTD/FTLD 50/119 (42.0)  AD 15/101 (14.9) | CB without physical aggression as this was analyzed in Liljegren et al. (2018a) already:  FTD/FTLD traffic violations > theft > sexual advances > mismanagement of personal finances;  AD traffic violations, theft & sexual advances | | | CB is more common in FTD/FTLD than in AD;  Higher amount of police interaction and recurrence of CB in FTD/FTLD than AD;  Non-tau pathology (fused in sarcoma, TDP 43) seems to be associated with CB in FTD/FTLD | JBI 7/9  NOS 6/9 |
| McDonell et al. (2021) | R | USA,  Medical records,  2006-2020 | N=282  127:155 | HD  (ICD-9 or ICD-10) | ICD-9 or ICD-10;  Patients medical notes | | HD without CB | NS | NS | HD 31/282 (11) | Physical violence > traffic violations > substance abuse > illegal financial activity > sexual advances | | | CB problem in HD, predominantly seen in males;  CB in HD associated with psychiatric comorbidity | JBI 8/9  NOS 4/9 |
| Mendez et al. (2005) | R | USA,  Medical records & caregiver interviews | N=56  28:28  62 (bvFTD)  66 (AD) | bvFTD  AD  (Neary et al. 1998 & McKhann et al. 1984) | MMSE, CDR,  PET/SPECT;  Caregivers interview for sociopathic acts that are potential grounds for arrest | | AD | NS | MMSE  23.6 (FTD)  21.3 (AD)  CDR  1.6 (FTD)  1.8 (AD) | bvFTD 16/28 (57)  AD 2/28 (7) | Sexual advances & traffic violations > physical assaults | | | Sociopathic acts are more common in bvFTD than AD;  Patients with bvFTD are aware of these acts, recognized them as wrong but could not prevent from behaving impulsively;  Sociopathic acts in bvFTD associated with impaired motor inhibition (alternate tapping, Go/No-Go) & right frontotemporal involvement on PET/SPECT | JBI 3/9  NOS 4/9 |
| Miller et al. (1997) | R | USA,  Medical records & caregiver interviews | N=44  19:25  FTD 61  AD 62 | FTD/AD  (Criteria by Lund & Manchester groups for FTD, Brun et al. 1994; criteria for probable AD by McKhann et al. 1984) | MRI, SPECT,  histopathology in 8/22 patients with clinical FTD (for all diagnosis confirmed); Medical records & caregiver interviews | | AD | NS | MMSE  22 (FTD)  19 (AD) | FTD 11/22 (50)  AD 1/22 (5) | Stealing > physical  assault, sexual comments & advances > hit and run accident > unethical job conduct, public urination & indecent exposure (not specified for FTD & AD) | | | Antisocial behaviors that might be regarded as CB much more frequent in FTD than AD;  Three subjects with FTD arrested, two prevented from arrest by relatives who convinced authorities that subject suffered from disease | JBI 7/9  NOS 5/9 |
| Miller et al. (2019) | R | USA,  Medical records,  1988-2012 & Records from South Carolina law enforcement division | N=141,281  80 | AD  (ICD-9) | ICD-9;  Records from South Carolina law enforcement division | | AD without DUI | NS | NS | AD 2882/141,281 (2) | Only arrests due to DUI analyzed | | | Arrests due to DUI associated with younger age at AD diagnosis and increased mortality after diagnosis;  Men with higher risk than women;  African-American higher risk than white registrants | JBI 7/9  NOS 5/9 |
| Mychack et al. (2001) | R | USA,  Medical records & caregiver interviews | N=41 | Right-sided bvFTD  (Neary et al. 1998) | MRI, SPECT to assess laterality;  Medical records & caregiver interviews to assess CB | | Left-sided bvFTD | NS | NS | 7/12 (58.3) of right-sided bvFTD  (NS for left-sided bvFTD) | NS, but socially undesirable behavior in right-sided bvFTD aggression > sexually deviant behavior > financial recklessness | | | Right sided bvFTD with more (11/12, 92%) frequent socially undesirable behavior as initial symptom (including CB) than in left-sided bvFTD (2/19, 11%) | JBI 3/9  NOS 4/9 |
| Phan et al. (2023) | P | USA,  Medical records & caregiver interviews,  2018-2021 | N=56  39:17  63 (bvFTD)  68 (other FTLD)  71 (AD/MCI) | bvFTD  PSP  svPPA  nfvPPA  AD/MCI  (Rascovsky et al. 2011, Gorno-Tempini et al. 2011, Höglinger et al. 2017, McKhann et al. 2011, Armstrong et al. 2013) | Medical records;  Caregiver interview | | AD/MCI | 2.5 (bvFTD)  2 (other FTD)  3 (AD/MCI) | NS | bvFTD 18/23 (78.3)  Other FTLD (PSP, svPPA & nfvPPA) 1/14 (7.1)  AD/MCI: 4/19 (21.1) | bvFTD Physical threat > harm > theft > traffic violations;  AD Physical harm > threat > traffic violations;  Other FTLD Theft | | | Prevalence (91%) and severity of antisocial behavior was significantly higher in bvFTD than in other dementias;  CB was more common in bvFTD than in other forms of dementia;  Social behavior related to disease duration and severity as well as bilateral frontomedian atrophy | JBI 7/9  NOS 5/9 |
| Shinagawa et al. (2017) | R | Japan,  Ten Nationwide specialized dementia outpatient clinics,  2011-2015 | N=412  180:232  67 (bvFTD)  70 (svPPA)  69 (AD) | bvFTD  svPPA  AD  (Rascovsky et al. 2011, Gorno-Tempini et al. 2011, McKhann et al. 1984) | MMSE  CDR;  Patients medical files | | AD (age and sex matched) | 4 (bvFTD)  5 (svPPA) | MMSE 18 | FTD 42/157 (27)  bvFTD 24/73 (33)  svPPA 18/84 (21)  AD 14/255 (6) | bvFTD theft > traffic > nuisance acts > violence > fare evasion > urination in public & trespassing;  svPPA theft > traffic > nuisance acts > urination in public > violence & fare evasion;  AD traffic > theft > trespassing & violence > urination in public | | | CB in bvFTD and svPPA (and FTD) higher than in AD;  CB decreased in all groups after initial consultation;  CB rate generally higher in men than women;  Higher disease severity (CDR) associated with more CB | JBI 7/9  NOS 4/9 |
| Talaslahti et al. (2021) | R | Finland,  Finnish  Hospital discharge register & Finnish Police register,  1998-2015 | N=92,191  33,294:58,897  71 (FTD)  81 (AD)  73 (LBD/PDD) | FTD  AD  LBD/PDD  (ICD-10) | ICD-10;  Police register | | Finnish population (age-matched) | -4 - 0 | NS | CB four years before diagnosis of dementia:  FTD 139/1060 (13.1)  AD 4366/80,540 (5.4)  LBD/PDD 784/10,591 (7.4) | Traffic offences > property > violence > other > alcohol & sexual | | | CB may manifest years before diagnosis of dementia;  More CB in men than women in all three dementias;  Higher CB rate in FTD than AD and LBD/PDD;  Male and female criminality rates in FTD and AD were higher than in the general population | JBI 8/9  NOS 7/9 |
| Talaslahti et al. (2023) | R | Finland,  Finnish Care Register for Health Care & Finnish National Police Register,  1998-2015 | N=92,191  33,294:58,897 | FTD  AD  LBD/PDD  (ICD-10) | ICD-10;  Police register | | Finnish population (age-matched) | -1 - 0 | NS | CB one year before diagnosis of dementia:  FTD 49/1060 (4.6)  AD 1244/80,540 (1.5)  LBD/PDD 229/10,591 (2.2) | Traffic offences > property > violence > other > alcohol > sexual | | | CB is more common in men than women in all three dementias;  Persons showing CB before dementia are younger than those without CB | JBI 8/9  NOS 7/9 |
| Trobe et al. (1996) | R | USA,  Medical records & Police records,  1986-1993 | N=143  67:76  71 | AD | MMSE;  Driving records of the state | | Population (age-matched) | NS | MMSE 14.8 | AD 33/143 (23)  Control 156/715 (22) | Only car crashes analyzed | | | AD patients and general population do not differ in amount of crashes,  neither before nor after diagnosis | JBI 5/9  NOS 5/9 |

Note: for the quality score, some sections were not applicable (NA) for the study, but NA was counted as 0 in the score.

Abbreviations: AD Alzheimer’s disease, bvFTD behavioral variant FTD, CB criminal behavior, CBS corticobasal syndrome, CDR Clinical Dementia Rating scale, DUI driving under influence of alcohol, FTD frontotemporal dementia, FTLD frontotemporal lobar degeneration (histopathological diagnosis, including bvFTD, svPPA, nfvPPA, PSP, corticobasal degeneration), HD Huntington’s disease, ICD International Classification of Diseases, JBI Joanna Briggs Institute checklist, LBD Lewy body dementia, lvPPA logopenic variant PPA, MCI mild cognitive impairment, MD mixed dementia (AD and VaD), MMSE Mini Mental State Examination, MRI magnetic resonance imaging, N number, nfvPPA non-fluent agrammatic variant PPA, NOS Newcastle-Ottawa Scale checklist, NS not specified, PDD Parkinson’s disease dementia, PET positron emission tomography, PPA primary progressive aphasia, PSP progressive supranuclear palsy, R retrospective, P prospective, SPECT single photon emission computer tomography, svPPA semantic variant PPA, TDP-43 transactive response DNA-binding protein 43, VaD vascular dementia.

References for studies included in meta-analysis & diagnostic criteria

1. Armstrong, M. J., Litvan, I., Lang, A. E., Bak, T. H., Bhatia, K. P., Borroni, B., Boxer, A. L., Dickson, D. W., Grossman, M., Hallett, M., Josephs, K. A., Kertesz, A., Lee, S. E., Miller, B. L., Reich, S. G., Riley, D. E., Tolosa, E., Tröster, A. I., Vidailhet, M., & Weiner, W. J. (2013). Criteria for the diagnosis of corticobasal degeneration. Neurology, 80(5), 496–503. https://doi.org/10.1212/WNL.0b013e31827f0fd1.
2. Brun, A., Englund, B., Gustafson, L., Passant, U., Mann, D.M.A., Neary, D., Snowden, J.S. (1994). Clinical and neuropathological criteria for frontotemporal dementia. The Lund and Manchester Groups. J Neurol Neurosurg Psychiatry, 57(4), 416-8. doi: 10.1136/jnnp.57.4.416.
3. Diehl-Schmid, J., Ernst, J., Krapp, S., Förstl, H., Nedopil, N., & Kurz, A. (2006). Frontotemporale Demenz und delinquentes Verhalten [Misdemeanour in Frontotemporal Dementia]. Fortschritte Neurologie und Psychiatrie, 74, 203-210. https://doi.org/10.1055/s−2005−870962.
4. Diehl-Schmid, J., Perneczky, R., Koch, J., Nedopil, N., & Kurz, A. (2013). Guilty by suspicion? Criminal behavior in frontotemporal lobar degeneration. Cognitive and Behavioral Neurology, 26(2), 73-77. https://doi.org/10.1097/WNN.0b013e31829cff11
5. Ginters, M., Talaslahti, T., Palm, A., Kautiainen, H., Vataja, R., Elonheimo, H., Suvisaari, J., Lindberg, N., Koponen, H. (2023). Criminal behaviour after diagnosis of a neurocognitive disorder: A nationwide Finnish register study. American Journal of Geriatric Psychiatry, 31(8), 598-606. doi: 10.1016/j.jagp.2023.01.025.
6. Gorno-Tempini, M. L., Hillis, A. E., Weintraub, S., Kertesz, A., Mendez, M., Cappa, S. F., Ogar, J. M., Rohrer, J. D., Black, S., Boeve, B. F., Manes, F., Dronkers, N. F., Vandenberghe, R., Rascovsky, K., Patterson, K., Miller, B. L., Knopman, D. S., Hodges, J. R., Mesulam, M. M., & Grossman, M. (2011). Classification of primary progressive aphasia and its variants. Neurology, 76(11), 1006–1014. https://doi.org/10.1212/WNL.0b013e31821103e6.
7. Höglinger, G. U., Respondek, G., Stamelou, M., Kurz, C., Josephs, K. A., Lang, A. E., Mollenhauer, B., Müller, U., Nilsson, C., Whitwell, J. L., Arzberger, T., Englund, E., Gelpi, E., Giese, A., Irwin, D. J., Meissner, W. G., Pantelyat, A., Rajput, A., Van Swieten, J. C., Troakes C, Antonini A, Bhatia KP, Bordelon Y, Compta Y, Corvol JC, Colosimo C, Dickson DW, Dodel R, Ferguson L, Grossman M, Kassubek J, Krismer F, Levin J, Lorenzl S, Morris HR, Nestor P, Oertel WH, Poewe W, Rabinovici G, Rowe JB, Schellenberg GD, Seppi K, van Eimeren T, Wenning GK, Boxer AL, Golbe LI, Litvan I; Movement Disorder Society-endorsed PSP Study Group. (2017). Clinical diagnosis of progressive supranuclear palsy: The movement disorder society criteria: MDS Clinical Diagnostic Criteria for PSP. Movement Disorders, 32(6), 853–864. https://doi.org/10.1002/mds.26987.
8. Liljegren, M., Naasan, G., Temlett, J., Perry, D. C., Rankin, K. P., Merrilees, J., Grinberg, L. T., Seeley, W. W., Englund, E., & Miller, B. L. (2015). Criminal behavior in frontotemporal dementia and Alzheimer disease. JAMA Neurology, 72(3), 295-300. https://doi.org/10.1001/jamaneurol.2014.3781.
9. Liljegren, M., Landqvist Waldö, M., & Englund, E. (2018a). Physical aggression among patients with dementia, neuropathologically confirmed post-mortem. International Journal of Geriatric Psychiatry, 33(2), e242-e248. doi: 10.1002/gps.4777.
10. Liljegren, M., Landqvist Waldö, M., Rydbeck, R., & Englund, E. (2018b). Police interactions among neuropathologically confirmed dementia patients: Prevalence and cause. Alzheimer Disease and Associated Disorders, 32(4), 346-350. https://doi.org/10.1097/WAD.0000000000000267.
11. Liljegren, M., Landqvist Waldö, M., Frizell Santillo, A., Ullén, S., Rydbeck, R., Miller, B., & Englund, E. (2019). Association of neuropathologically confirmed frontotemporal dementia and Alzheimer disease with criminal and socially inappropriate behavior in a Swedish cohort. JAMA Network Open, 2(3), e190261. https://doi.org/10.1001/jamanetworkopen.2019.0261.
12. McDonell, K. E., Brown, B. K., Hale, L., Darby, R. R., Stovall, J., Compas, B. E., & Claassen, D. O. (2021). Medicolegal aspects of Huntington disease. The Journal of the American Academy of Psychiatry and the Law, 49(4), 565-571. https://doi.org/10.29158/JAAPL.210008-21.
13. McKhann, G., Drachman, D., Folstein, M., Katzman, R., Price, D., Stadlan, E.M. (1984) Clinical diagnosis of Alzheimer’s disease: Report of the NINCDS-ADRDA Work Group under the auspices of Department of Health and Human Services Task Force on Alzheimer’s Disease. Neurology, 34, 939-944.
14. McKhann, G. M., Knopman, D. S., Chertkow, H., Hyman, B. T., Jack, C. R., Kawas, C. H., Klunk, W. E., Koroshetz, W. J., Manly, J. J., Mayeux, R., Mohs, R. C., Morris, J. C., Rossor, M. N., Scheltens, P., Carrillo, M. C., Thies, B., Weintraub, S., & Phelps, C. H. (2011). The diagnosis of dementia due to Alzheimer’s disease: Recommendations from the National Institute on Aging‐Alzheimer’s Association workgroups on diagnostic guidelines for Alzheimer’s disease. Alzheimer’s & Dementia, 7(3), 263–269. https://doi.org/10.1016/j.jalz.2011.03.005.
15. Mendez, M. F., Chen, A. K., Shapira, J. S., Miller, B. L. (2005). Acquired sociopathy and frontotemporal dementia. Dementia and Geriatric Cognitive Disorders, 20(2–3), 99-104. https://doi.org/10.1159/000086474.
16. Miller, B. L., Darby, A., Benson, D. F., Cummings, J. L., & Miller, M. H. (1997). Aggressive, socially disruptive and antisocial behaviour associated with fronto-temporal dementia. British Journal of Psychiatry, 170(2), 150–155. https://doi.org/10.1192/bjp.170.2.150.
17. Miller, M., Orwat, D., Rahimi, G., Mintzer, J. (2019). A retrospective, population-based cohort study of driving under the influence, Alzheimer's disease diagnosis, and survival. Int Psychogeriatr, 31(4), 571-577. doi: 10.1017/S1041610218001151.
18. Mychack, P., Kramer, J. H., Boone, K. B., & Miller, B. L. (2001). The influence of right frontotemporal dysfunction on social behavior in frontotemporal dementia. Neurology, 56(11 Suppl 4), S11-15. https://doi.org/10.1212/wnl.56.suppl_4.s11
19. Neary, D., Snowden, J. S., Gustafson, L., Passant, U., Stuss, D., Black, S., Freedman, M., Kertesz, A., Robert, P. H., Albert, M., Boone, K., Miller, B. L., Cummings, J., & Benson, D. F. (1998). Frontotemporal lobar degeneration: A consensus on clinical diagnostic criteria. Neurology, 51(6), 1546–1554. https://doi.org/10.1212/WNL.51.6.1546
20. Phan, T. X., Reeder, J.E., Keener, L.C., Considine, C.M., Zald, D.H., Claassen, D.O., Darby, R.R. (2023). Measuring antisocial behaviors in behavioral variant frontotemporal dementia with a novel informant-based questionnaire. Journal of Neuropsychiatry and Clinical Neurosciences, 35(4), 374-384. doi: 10.1176/appi.neuropsych.20220135.
21. Rascovsky, K., Hodges, J. R., Knopman, D., Mendez, M. F., Kramer, J. H., Neuhaus, J., van Swieten, J. C., Seelaar, H., Dopper, E. G. P., Onyike, C. U., Hillis, A. E., Josephs, K. A., Boeve, B. F., Kertesz, A., Seeley, W. W., Rankin, K. P., Johnson, J. K., Gorno-Tempini, M.-L., Rosen, H., … Miller, B. L. (2011). Sensitivity of revised diagnostic criteria for the behavioural variant of frontotemporal dementia. Brain, 134(9), 2456–2477. https://doi.org/10.1093/brain/awr179.
22. Shinagawa, S., Shigenobu, K., Tagai, K., Fukuhara, R., Kamimura, N., Mori, T., Yoshiyama, K., Kazui, H., Nakayama, K., & Ikeda, M. (2017). Violation of laws in frontotemporal dementia: A multicenter study in Japan. Journal of Alzheimer’s Disease, 57(4), 1221–1227. https://doi.org/10.3233/JAD-170028.
23. Talaslahti, T., Ginters, M., Kautiainen, H., Vataja, R., Elonheimo, H., Erkinjuntti, T., Suvisaari, J., Lindberg, N., & Koponen, H. (2021). Criminal behavior in the four years preceding diagnosis of neurocognitive disorder: A nationwide register study in Finland. The American Journal of Geriatric Psychiatry, 29(7), 657-665. https://doi.org/10.1016/j.jagp.2020.11.011.
24. Talaslahti, T., Ginters, M., Kautiainen, H., Vataja, R., Palm, A., Elonheimo, H., Suvisaari, J., Lindberg, N., & Koponen, H. (2023). Crime, mortality and neurocognitive disorders: A nationwide register study in Finland. International Journal of Methods in Psychiatric Research, 32(2), e1948. https://doi.org/10.1002/mpr.1948.
25. Trobe, J.D., Waller, P.F., Cook-Flannagan, C.A., Teshima, S.M., Bieliauskas, L.A. (1996). Crashes and violations among drivers with Alzheimer disease. Arch Neurol, 53(5), 411-6. doi: 10.1001/archneur.1996.00550050033021.

Table S2 Number of subjects with and without criminal behavior plus prevalence measures for the different cohorts with neurodegenerative and dementia syndromes


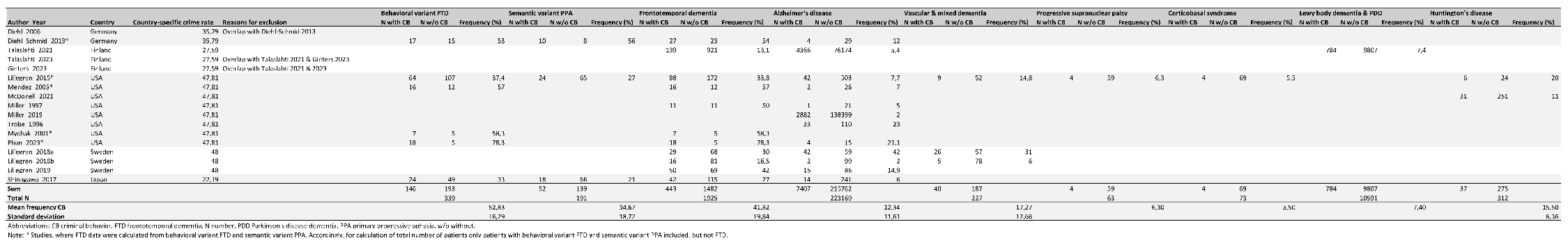


Figure S1 Results for odds ratio analysis

| FTD vs. AD | |
| --- | --- |
| 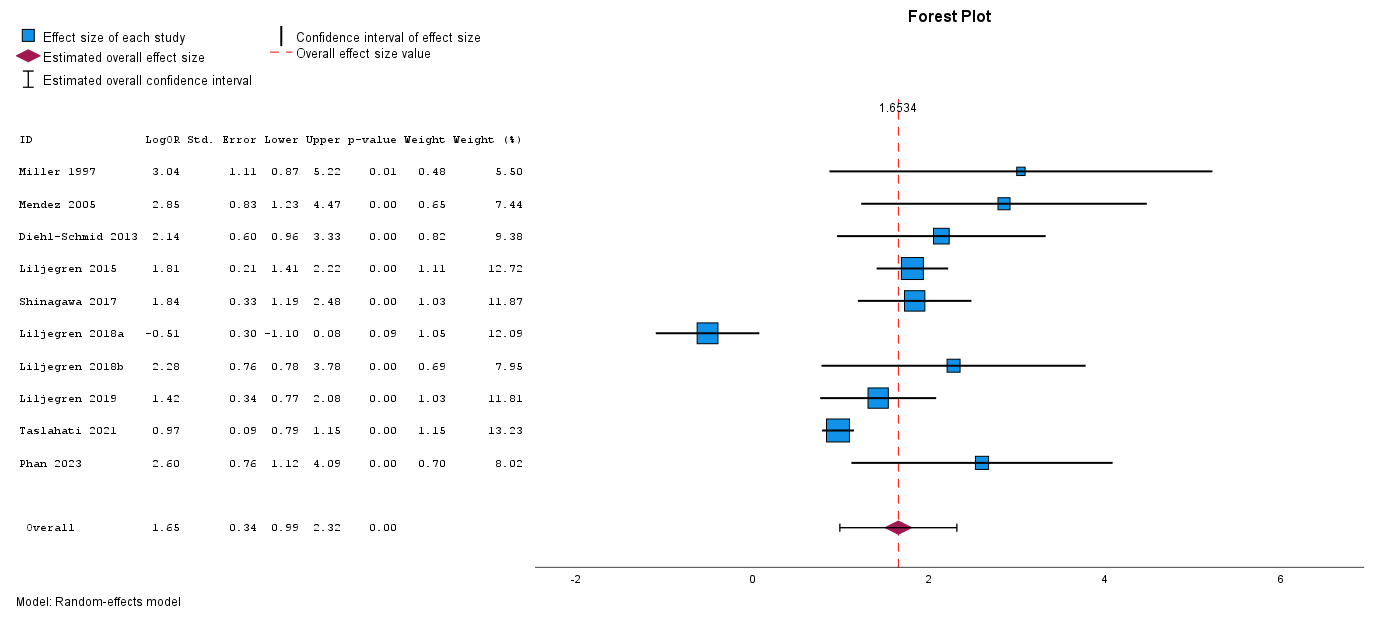 | 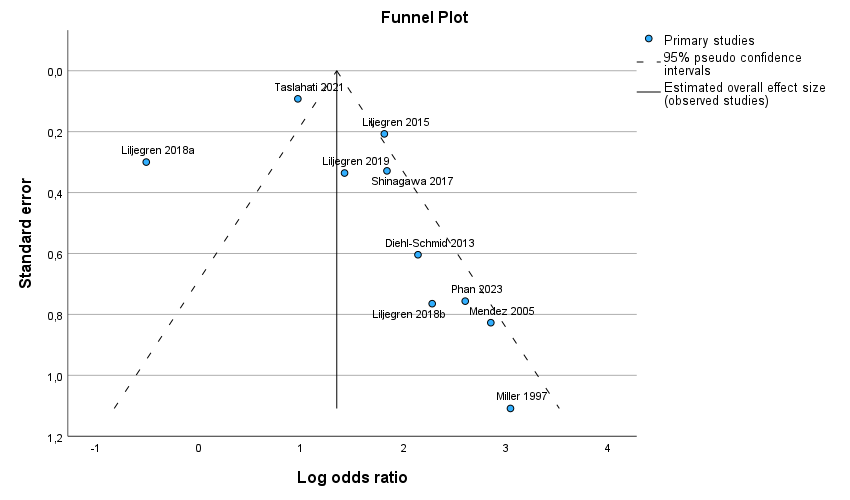 |
| bvFTD vs. AD | |
| 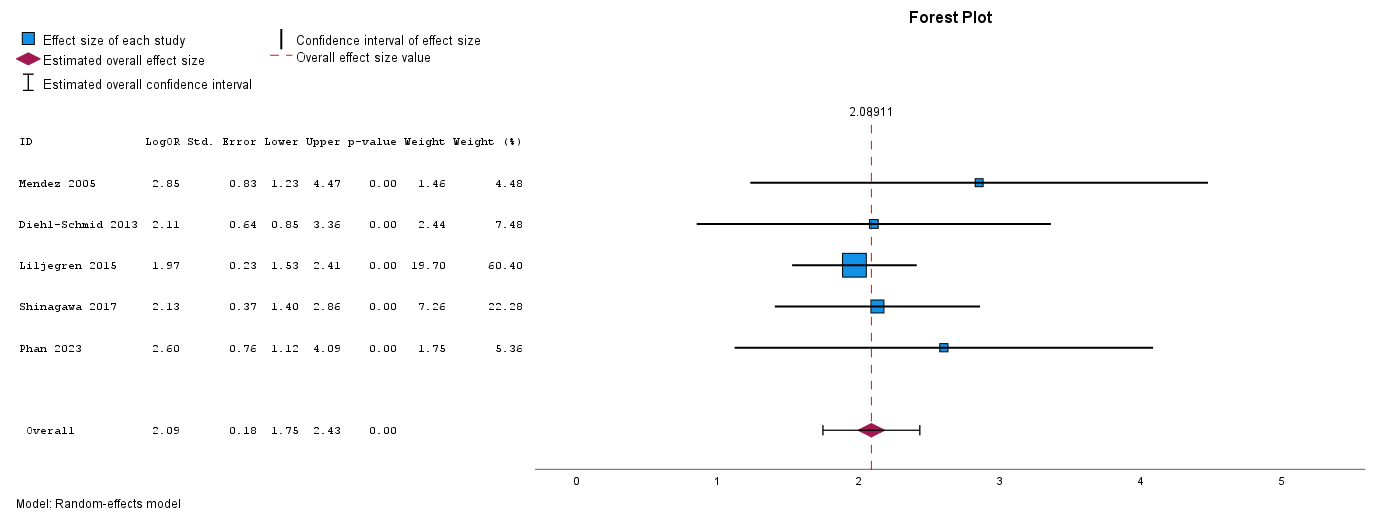 | 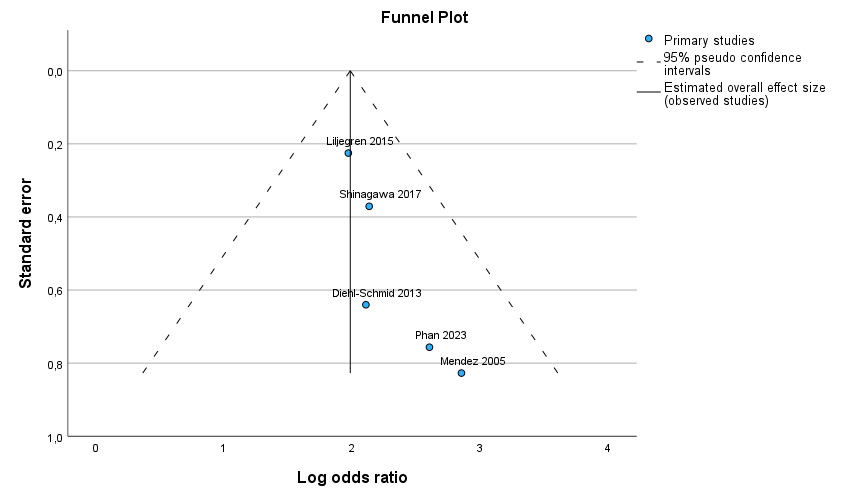 |

| svPPA vs. AD | |
| --- | --- |
| 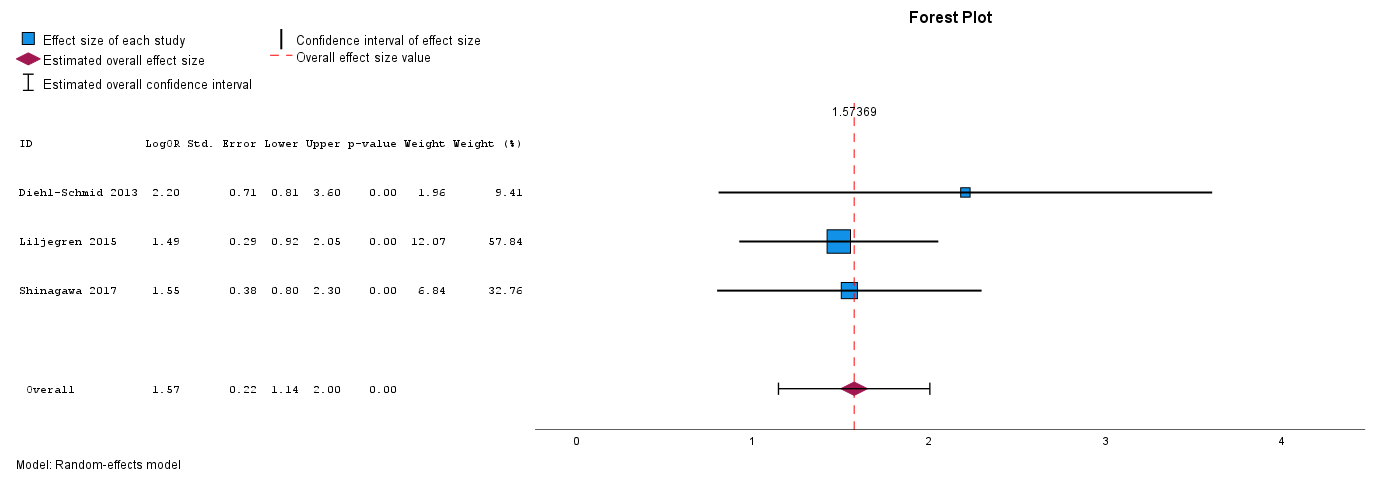 | 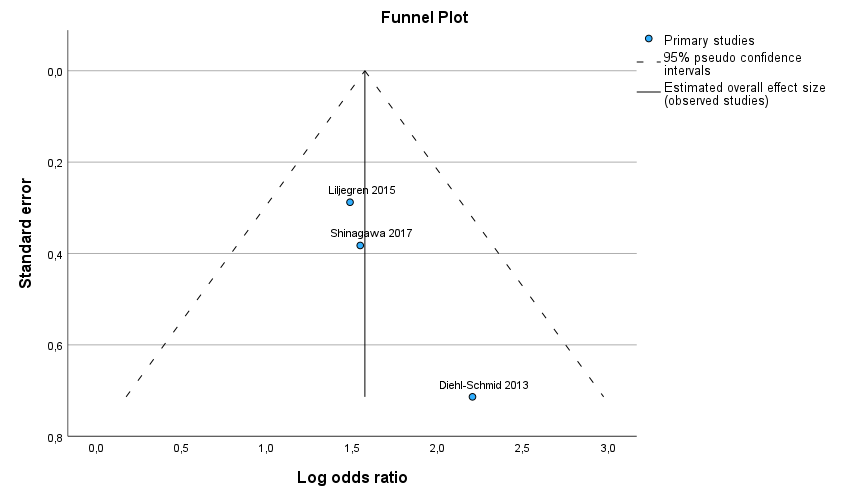 |
| bvFTD vs. svPPA | |
| 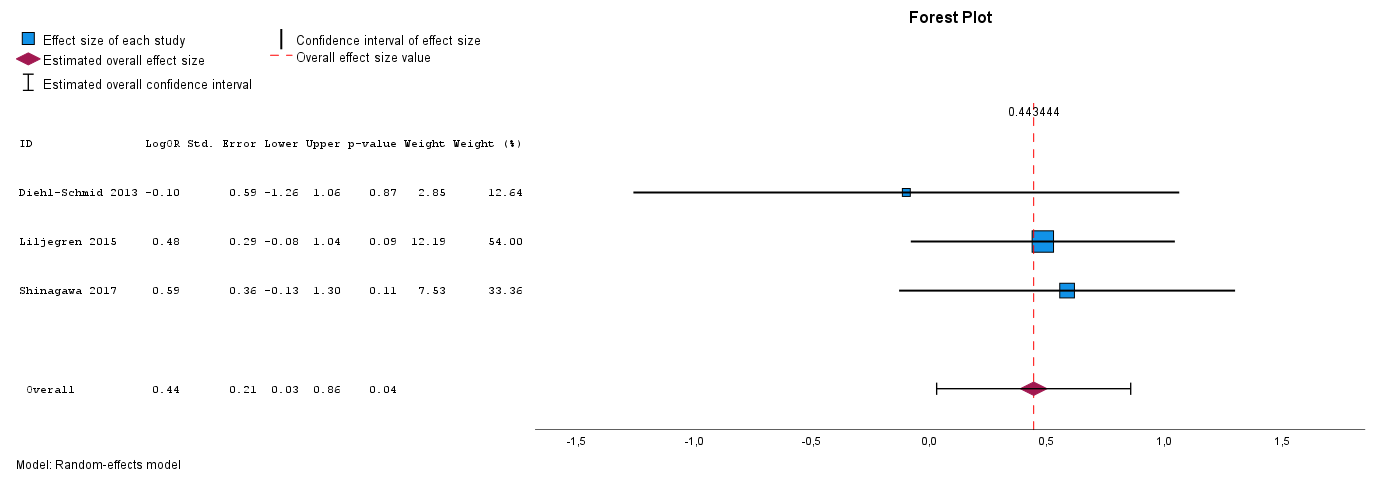 | 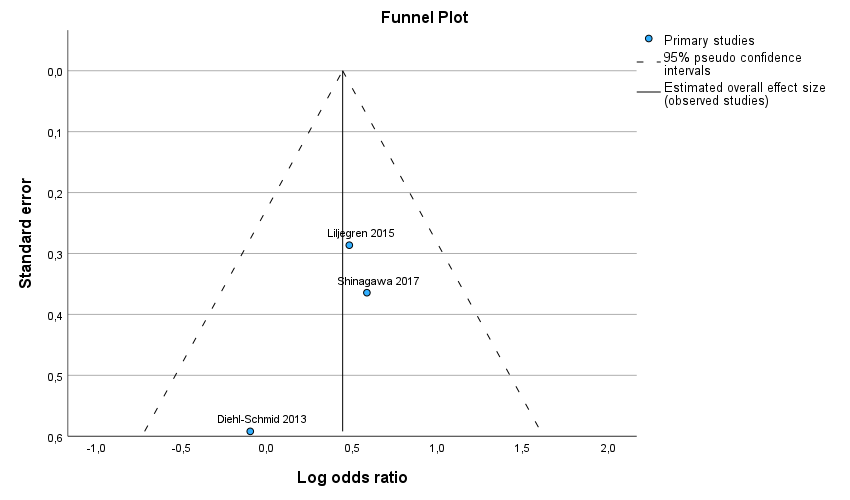 |

| FTD vs. VaD/MD | |
| --- | --- |
| 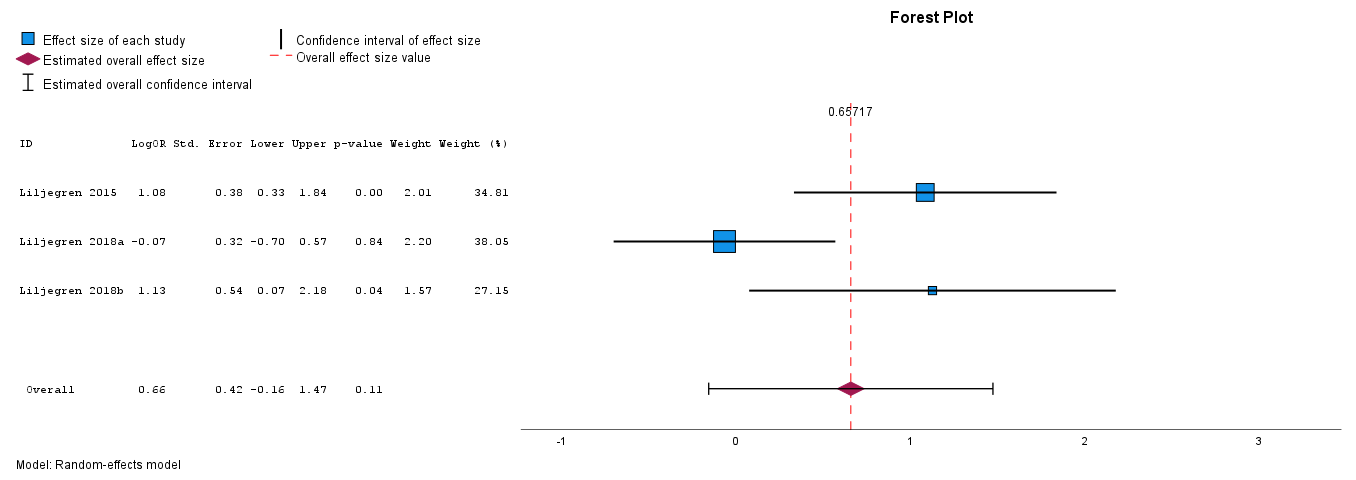 | 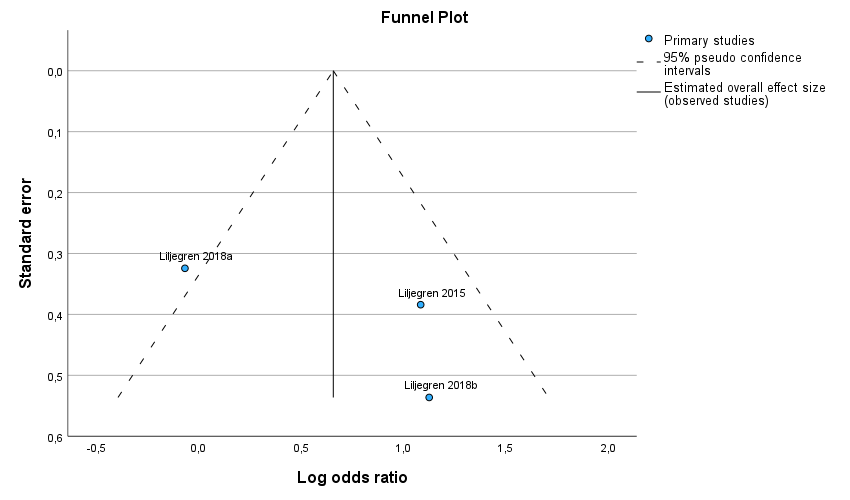 |
| AD vs. VaD/MD | |
| 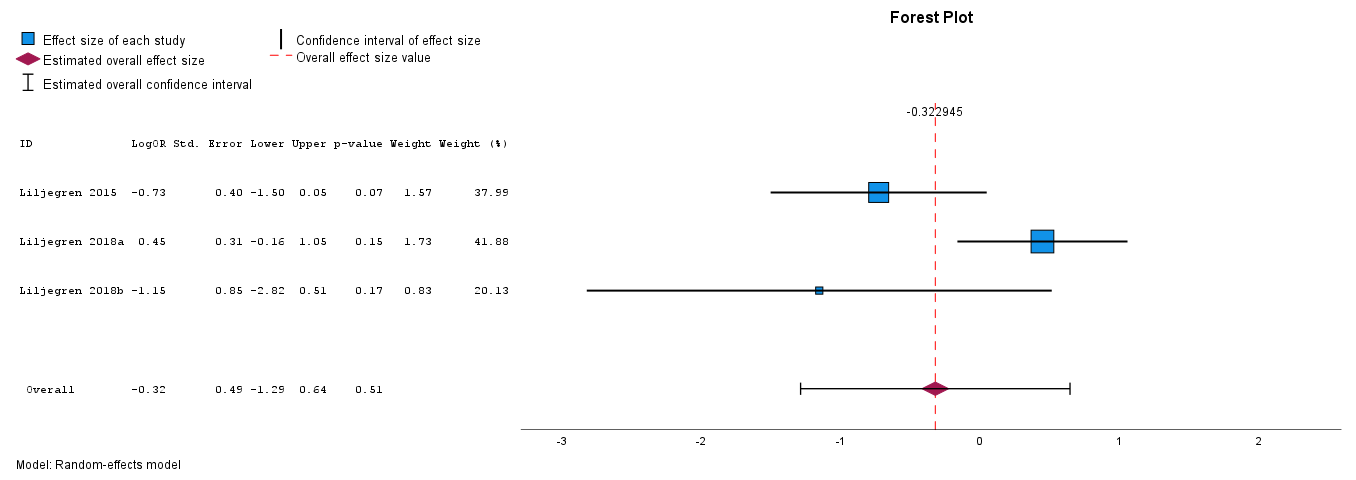 | 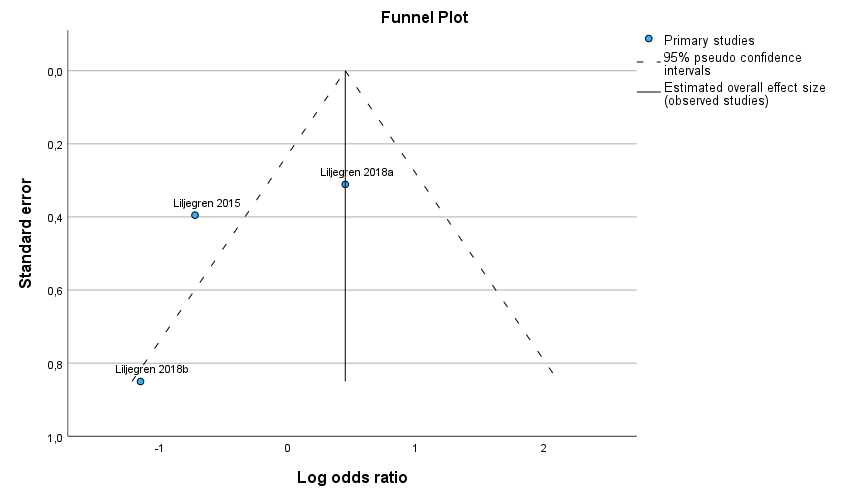 |

| FTD vs. ParkS | |
| --- | --- |
| 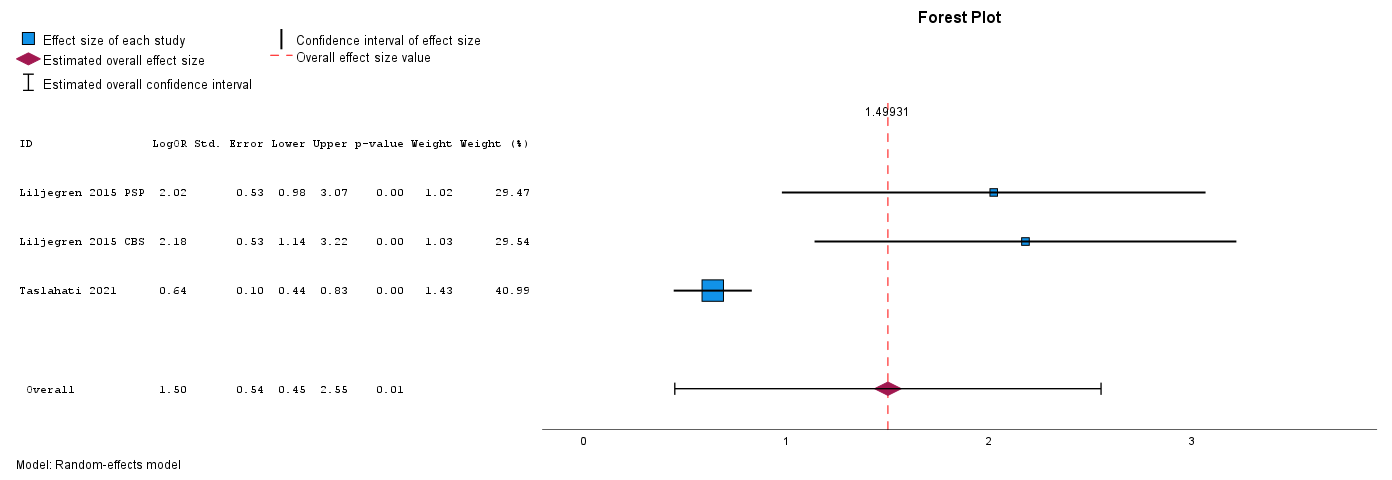 | 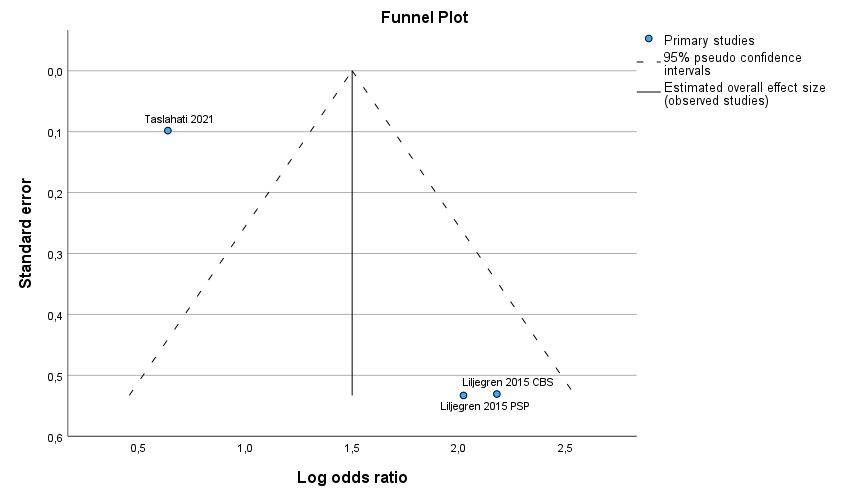 |
| AD vs. ParkS | |
| 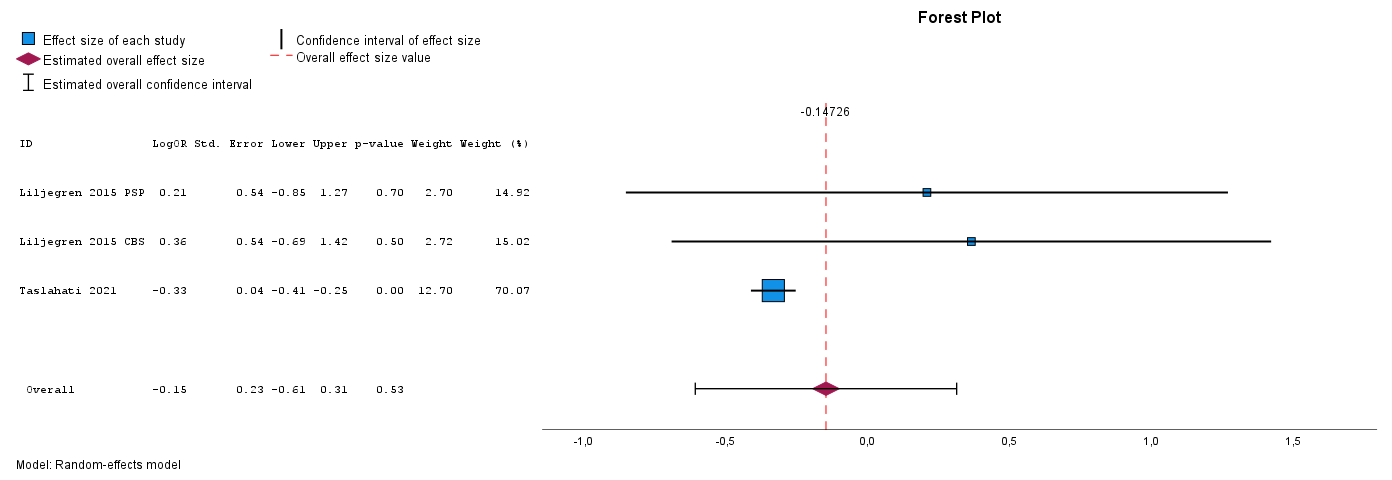 | 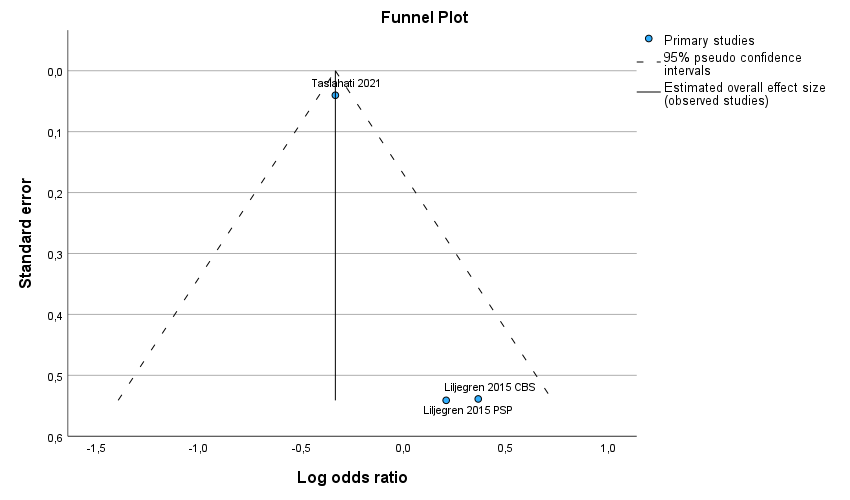 |

| FTD vs. AD without Liljegren et al. 2018a and Taslahati et al. 2021 | |
| --- | --- |
| 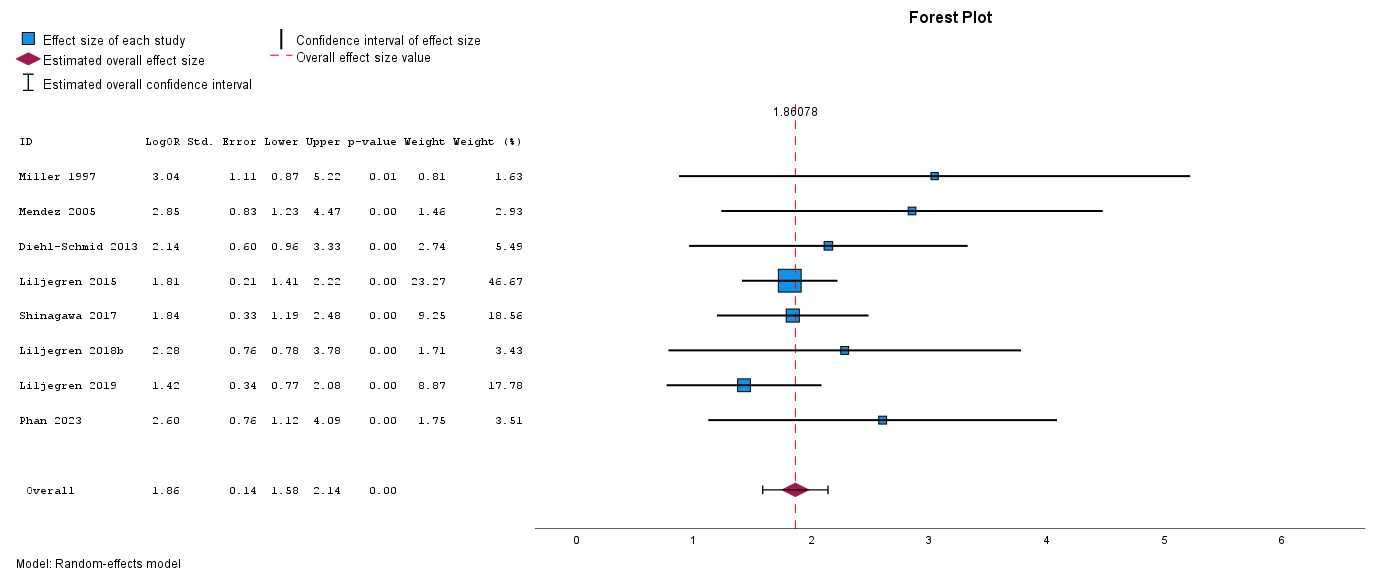 | 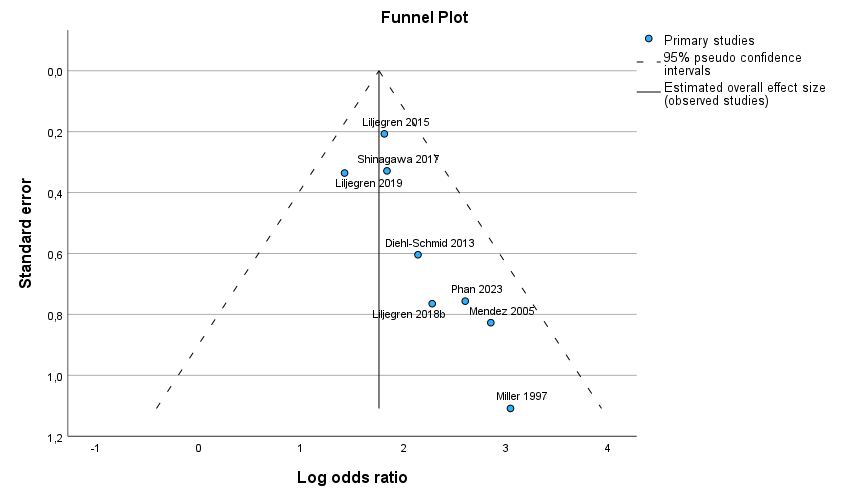 |
